# [^186^Re]Re- and [^99m^Tc]Tc-Tricarbonyl Metal Complexes with 1,4,7-Triazacyclononane-Based Chelators Bearing Amide, Alcohol, or Ketone Pendent Groups

**DOI:** 10.1021/acsomega.4c05699

**Published:** 2024-09-11

**Authors:** Rebecca Hoerres, Ritin Kamboj, Nora Pryor, Steven P. Kelley, Heather M. Hennkens

**Affiliations:** † Department of Chemistry, University of Missouri, 601 South College Avenue, Columbia, Missouri 65211, United States; ‡ Research Reactor Center, University of Missouri, 1513 Research Park Drive, Columbia, Missouri 65211, United States

## Abstract

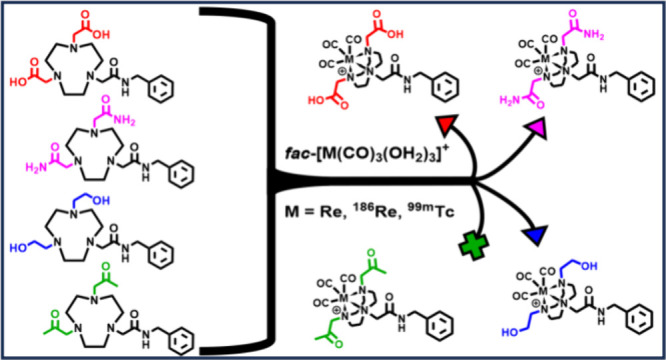

1,4,7-Triazacyclononane (TACN)-based chelators, such as NOTA and NODAGA, have shown great promise as bifunctional chelators for [M(CO)_3_]^+^ cores (M = ^99m^Tc and ^186^Re) in radiopharmaceutical development. Previous investigations of TACN-based chelators bearing pendent acid and ester arms demonstrated the important role the pendent arms have in successful coordination of the [M(CO)_3_]^+^ core with the TACN backbone nitrogens. In this work, we introduce three TACN-based bifunctional chelators bearing amide, alcohol, and ketone pendent arms and evaluate their (radio)labeling efficiency with the [M(CO)_3_]^+^ core as well as the *in vitro* stability and hydrophilicity of the resulting radiometal complexes. Following their synthesis and characterization, the amide (**2**) and alcohol (**3**) chelators were successfully labeled with the [M(CO)_3_]^+^ cores (M = ^nat^Re, ^99m^Tc, and ^186^Re), while the ketone (**4**) was not successfully labeled. Radiometal complexes **M-2** and **M-3** demonstrated hydrophilic character in logD_7.4_ studies as well as excellent stability in phosphate-buffered saline (pH 7.4), l-histidine, l-cysteine, and rat serum at 37 °C through 24 h. While the hydrophilicity and stability of these radiocomplexes are attractive, future TACN chelator design modifications to increase radiolabeling yields under milder reaction conditions would improve their potential for use in development of [M(CO)_3_]^+^ radiopharmaceuticals.

## 1 Introduction

The field of radiopharmaceutical development is growing exponentially as researchers and clinicians continue to demonstrate the effectiveness of using radiolabeled drugs for both diagnosis and treatment of diseases, such as cancer.^1−3^ Targeted radionuclide therapy (TRT) is of particular interest for the treatment of cancer because of its superior specificity for diseased cells over other treatments like chemotherapy and external beam radiation therapy.^4,5^ TRT commonly involves either the direct radiolabeling of a biological targeting vector,^6^ such as a peptide or antibody, or the conjugation of the biological targeting vector to a bifunctional chelator that is then radiolabeled with a suitable radionuclide.^7^ One example of the TRT approach is [^177^Lu]Lu-DOTATATE (Lutathera), which incorporates a radionuclide (^177^Lu), bifunctional chelator (DOTA), and targeting peptide (Tyr^3^-octreotate). Lutathera has been approved by both the Food and Drug Administration and the European Medicines Agency^8^ and has shown great success in the clinic for patients with gastroenteropancreatic neuroendocrine tumors. New developments in TRT are imperative to bring this success to patients with other types of cancer.

The use of bifunctional chelators allows for development of theranostic radiopharmaceuticals. Theranostic radiopharmaceuticals, as the name suggests, can be used for both therapy and diagnosis (imaging) of a disease. An identical pharmaceutical scaffold can be radiolabeled with either a radionuclide that decays by particle emission (e.g., alpha or beta minus decay) for therapeutic applications or a radionuclide that decays with photon emission (e.g., isomeric transition or positron decay) for diagnostic applications. The radionuclide(s) can be a single radionuclide that decays with both particle and photon emissions (e.g., ^64^Cu, positron and beta minus emissions), a pair of isotopes of the same element (e.g., ^155^Tb, imageable gamma emission, and ^161^Tb, beta minus emission), or a matched pair of radionuclides of different elements (e.g., ^99m^Tc, imageable gamma emission, and ^186/188^Re, beta minus emission). Ideally, if isotopes of two different elements are used as a theranostic matched pair, they will have similar chemical properties allowing for coordination to the same bifunctional chelator and a similar pharmacokinetic profile of the theranostic radiopharmaceuticals *in vivo*.

An example of a potential theranostic matched pair of radionuclides is ^99m^Tc and ^186^Re/^188^Re. In 2019, it was reported that of the 30 million nuclear medicine diagnostic scans conducted worldwide, 85% were performed using ^99m^Tc radiopharmaceuticals.^9^ Technetium-99m (*t*_1/2_ = 6.0 h) is extremely popular for diagnostic radiopharmaceutical applications because it is readily available in the form of a ^99^Mo/^99m^Tc generator, and it decays primarily by isomeric transition with the emission of a 140 keV gamma-ray that is ideal for single photon emission computed tomography (SPECT) imaging. Technetium has no nonradioactive isotopes or radioisotopes with useful therapeutic decay properties. However, rhenium is a congener of technetium with similar chemical and physical properties, making it the ideal candidate as a therapeutic counterpart. Two beta-minus emitting radioisotopes of rhenium, ^186^Re (*t*_1/2_ = 3.7 d) and ^188^Re (*t*_1/2_ = 17 h), have previously been explored as theranostic matched radionuclide partners to ^99m^Tc with promising results demonstrating similar pharmacokinetic profiles *in vivo*.^10,11^

Rhenium and technetium have rich redox chemistry, allowing them to bind to a variety of different bifunctional chelators. The +1 oxidation state of these metals can be accessed and stabilized through the synthesis of a [M(CO)_3_(OH_2_)_3_]^+^ precursor (M = ^99m^Tc, ^nat^Re, ^186^Re, and ^188^Re) from the permetallate or metal pentacarbonyl bromide chemical forms. The syntheses of these precursors are well established in literature.^12−14^ The [M(CO)_3_(OH_2_)_3_]^+^ precursors can be reacted with bifunctional chelators, during which the labile water ligands are replaced to form metal complexes. The tridentate NOTA chelator (2,2′,2″-(1,4,7-triazacyclononane-1,4,7-triyl)triacetic acid) has proven to be an excellent bifunctional chelator for coordination of the [M(CO)_3_]^+^ core (M = ^99m^Tc, ^nat^Re, and ^186^Re) due, in part, to the ionizable pendent acid arms that aid in (radio)labeling and increase the hydrophilicity of the overall metal complex.^15,16^ These [M(CO)_3_NOTA]^−^ metal complexes, both as model complexes and as peptide bioconjugates, have demonstrated excellent *in vitro* stability with moderate to high (radio)labeling yields.^10,15,16^

Our current work is investigating how changing the pendent arms on the 1,4,7-triazacyclononane (TACN) backbone impacts the chelator’s interaction with the [M(CO)_3_]^+^ core (M = ^99m^Tc, ^nat^Re, and ^186^Re). Modified TACN chelators have been evaluated with radionuclides including ^64^Cu and ^68^Ga,^17−19^ but there are very few examples of these chelators (radio)labeled with the [M(CO)_3_]^+^ cores.^15,20^ Previously, we evaluated TACN derivative chelators bearing no pendent arms, acid arms (NOTA derivative), ester arms, and mixed acid/ester arms.^16^ In those studies, we demonstrated that although the TACN pendent arms did not participate in coordination to the metal in the final complex, they played a significant role in facilitating (radio)labeling. Only the TACN chelator with no pendent arms and the chelators bearing at least one acid arm were successfully (radio)labeled with the [M(CO)_3_]^+^ cores. We hypothesized that there is an electrostatic attraction between the ionized pendent arms and the positively charged metal center, as well as a steric component, contributing to the success of the (radio)labeling. Further investigations into the interactions between the [M(CO)_3_]^+^ core and TACN-based chelators may provide insights to improve upon the design of these chelators for radiopharmaceutical applications.

With that in mind, this work presents three TACN-based chelators, namely, those bearing amide, alcohol, and ketone pendent arms, to evaluate how effectively they can be (radio)labeled with the [M(CO)_3_]^+^ cores. In this fundamental chemistry study, the amide and alcohol chelators were selected because they are not ionized in aqueous solutions at physiological pH; however, the polar functional groups they bear carry a partial negative charge that may directly or indirectly (e.g., via hydrogen bond formation with anions in solution) facilitate metal coordination. The ketone chelator was chosen as a comparator to the ester derivatives studied previously, as it does not contain a terminal polar group, but it is less sterically hindering than an ester group. TACN-based chelators bearing amide^21^ and alcohol^22^ pendent arms have been evaluated in the literature with gallium and zinc, respectively, with some success, but these chelators have not been evaluated with [M(CO)_3_]^+^ cores.

## 2 Materials and Methods

### 2.1 General

All chemicals were purchased from Sigma-Aldrich (St. Louis, Missouri) or Fisher Scientific (Pittsburgh, Pennsylvania) and were of reagent grade. The 1,4,7-triazacyclononane and *di*-*tert*-butyl 2,2′-(1,4,7-triazacyclononane-1–4-diyl)diacetate (NO2AtBu) were purchased from CheMatech (Dijon, France). Microwave syntheses were performed in a CEM Discover microwave synthesizer at a fixed power of 200 W.

Shimadzu Nexera or Prominence HPLCs with photodiode array (PDA) detectors and in-line NaI(Tl) detectors were used for HPLC analyses. The HPLC-grade solvents were filtered and degassed prior to use. All HPLC analyses were carried out on Thermo Fisher Scientific BetaBasic C18 columns (150 mm × 4.6 mm, 5 μm) with a flow rate of 1 mL/min and analyzed at 210 nm (TACN chelators) or 254 nm (Re-labeled chelators) wavelengths. Compound purifications were carried out on a Shimadzu Nexera semipreparative HPLC using a Phenomenex Luna C18 column (250 × 10 mm, 10 μm) with a flow rate of 4 mL/min. Binary linear gradients were used with pump A containing water (0.1% trifluoroacetic acid (TFA)) and pump B containing methanol (0.1% TFA). The following gradients were used for HPLC analyses and purifications: 5 to 95% B in A over 18 min (method 1), 30 to 55% B in A over 10 min (method 2), 30 to 40% B in A over 10 min (method 3), 30 to 80% B in A over 20 min (method 4), and 50 to 70% B in A over 10 min (method 5). Radio thin layer chromatography (radio-TLC) experiments were conducted on A81-24 saturation pads (Analtech, Newark, Delaware) and developed in 75% acetonitrile in saline. The radio thin layer chromatograms were read and analyzed on an Eckert and Ziegler AR-2000 radio-TLC Imager Scanner (Hopkinton, Massachusetts).

High-resolution electrospray ionization mass spectrometry (HS-ESI-MS) analyses were conducted on an LTQ Orbitrap XL or a Bruker timTOF-Pro2 mass spectrometer and analyzed with Xcalibur Qual Browser version 2.2 or CompassDA software. Liquid chromatography electrospray ionization mass spectrometry (LC-ESI-MS) analyses were conducted using a HPLC Gold System (Beckman Coulter, Fullerton, California) coupled to an ion trap mass spectrometer (LCQ Fleet from Thermo Fisher). The column, a Thermo Fisher BetaBasic C18 column (150 mm × 4.6 mm, 5 μm), was used with a flow rate of 1 mL/min and a binary linear gradient with pump A containing water (0.1% TFA) and pump B containing acetonitrile (0.1% TFA). The following gradient was used for LCMS analyses: 10 to 50% B in A over 30 min (method 6).

^1^H and ^13^C NMR spectra were recorded on a Bruker Avance III 500 or 600 MHz spectrometer and analyzed with Bruker TopSpin version 4.0.9. A Thermo Scientific Nicolet Summit Pro FTIR spectrometer was used for infrared spectroscopy analysis. Wavelengths between 500 and 4000 cm^–1^ were recorded. Combustion analysis was used to determine the elemental composition of the ^nat^Re-labeled complexes (Atlantic Microlab, Norcross, Georgia).

[^99m^Tc]TcO_4_^–^ in saline was obtained in high specific activity from ^99^Mo/^99m^Tc generators (Curium, St. Louis, Missouri) that were generously donated by Mid-America Isotopes, Inc. (Ashland, Missouri). The ^99m^Tc activity was measured by a Capintec CRC-55tR dose calibrator (Ramsey, New Jersey) or an ORTEC 4890 NaI(Tl) well detector. Low specific activity [^186^Re]ReO_4_^–^ (44–56 GBq/mg, 1.2–1.5 Ci/mg at the end of irradiation) was produced by neutron irradiation of enriched [^185^Re]Al(ReO_4_)_3_ targets at the University of Missouri Research Reactor (MURR). The ^186^Re activity was measured by a Capintec dose calibrator or an ORTEC HPGe GEM20–70 high-purity germanium detector (Oak Ridge, Tennessee) coupled to a Canberra multichannel analyzer (Meriden, Connecticut). All HPGe spectroscopy data were analyzed using the Canberra Genie 2000 (3.3) software.

### 2.2 *N*-Benzyl-2-(1,4,7-triazonan-1-yl)acetamide (**1**)

**Scheme 1:**
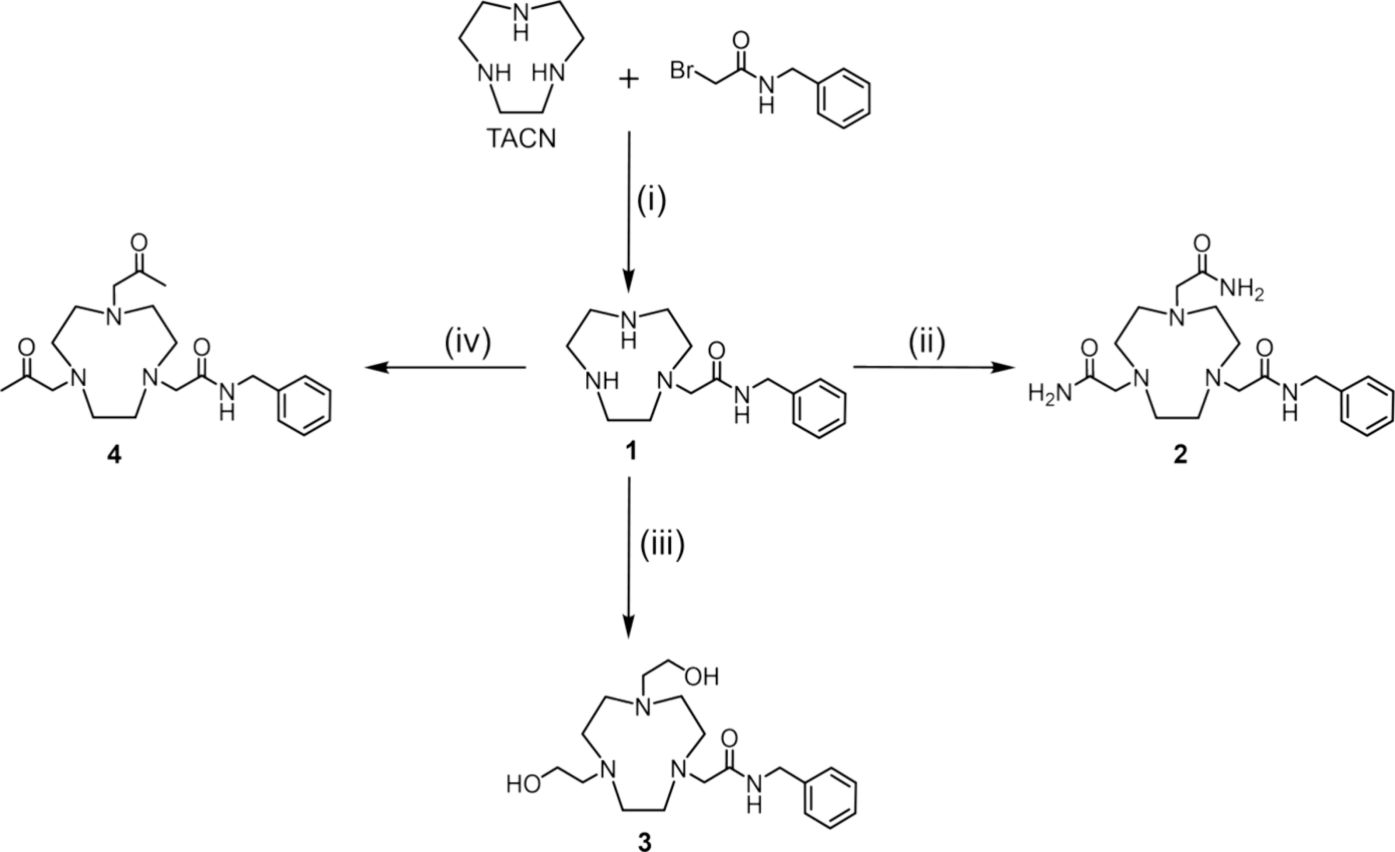
Synthesis of TACN-Based Chelators **2**–**4** (i) Potassium carbonate (1.5 equiv), acetonitrile, RT, 24 h, 40%. (ii) 2-Bromoacetamide (2 equiv), triethylamine (3 equiv), acetonitrile, 80 °C, 3 h, 74%. (iii) 2-Bromoethanol (2 equiv), triethylamine (3 equiv), acetonitrile, 80 °C, 9 h, 54%. (iv) 2-Chloroacetone (2 equiv), triethylamine (3 equiv), acetonitrile, 80 °C, 3 h, 93%.

Compound **1** was synthesized using two different methods (Scheme 1 and Scheme S1 in the Supporting Information). Details for the alternative synthetic method and synthesis of *N*-benzyl-2-bromoacetamide were reported previously^16,23^ and can be found in the Supporting Information. Commercially available TACN (100 mg, 0.77 mmol) and potassium carbonate (160 mg, 1.16 mmol) were dissolved in 5 mL of acetonitrile. In another vial, *N*-benzyl-2-bromoacetamide (175 mg, 0.77 mmol) was dissolved in 2.5 mL of acetonitrile and then added dropwise to the TACN solution over 20 min. After complete addition of the *N*-benzyl-2-bromoacetamide, the reaction was stirred at room temperature for 24 h. The solvent was removed under reduced pressure, and the crude residue was purified by semipreparative HPLC in small batches (method 1, *t*_R_ = 10.4 min). Isolated yield: 85 mg (40%). The product was characterized by LC-ESI-MS and ^1^H NMR. LC-ESI-MS (*m*/*z*) calculated for C_15_H_24_N_4_O [M + H]^+^ 277.28, found 277.31 (method 6, *t*_R_ = 7.9 min). ^1^H NMR (D_2_O, 300 MHz): δ_H_ 7.23–7.15 (m, 5H), 4.35 (s, 2H), 3.51 (s, 2H), 3.24 (t, 8H, *J* = 5.7 Hz), 2.99 (t, 4H, *J* = 5.5 Hz) and matches values previously reported in literature.^23^

### 2.3 2,2′-(7-(2-(Benzylamino)-2-oxoethyl)-1,4,7-triazonane-1,4-diyl)diacetamide (**2**)

The synthesis of model chelators **2**–**4** is shown in Scheme 1. Compound **1** (110 mg, 0.4 mmol) was dissolved in 3 mL of anhydrous acetonitrile. Triethylamine (166 μL, 1.2 mmol) and 2-bromoacetamide (110 mg, 0.8 mmol) were added, and the resulting solution was heated at 80 °C for 3 h in an oil bath. Reaction progress was monitored by analytical HPLC (method 1, *t*_R_ = 8.0 min). Upon reaction completion, the solvent was removed under reduced pressure, and the product was purified by semipreparative HPLC in small batches (method 1, *t*_R_ = 8.2 min). Isolated yield: 115 mg (74%). The product was characterized by HR-ESI-MS, ^1^H NMR, and ^13^C NMR. HR-ESI-MS (*m*/*z*) calculated for C_19_H_30_N_6_O_3_ [M + H]^+^ 391.2452, found 391.2444. ^1^H NMR (CD_3_CN, 600 MHz): δ_H_ 7.66 (t, 1H, *J* = 5.8 Hz), 7.39–7.29 (m, 5H), 6.90 (s, 2H), 6.35 (s, 2H), 4.41 (d, 2H, *J* = 5.7 Hz), 3.73 (s, 2H), 3.68 (s, 2H), 3.13 (s, 12H). ^13^C NMR (CD_3_CN, 600 MHz): δ_C_ 171.1, 168.8, 138.4, 128.5, 127.6, 127.2, 58.0, 57.2, 50.4, 50.2, 43.0. Values were compared to and matched those reported in the literature.^24^

### 2.4 *N*-Benzyl-2-(4,7-bis(2-hydroxyethyl)-1,4,7,-triazonan-1-yl)acetamide (**3**)

Compound **1** (130 mg, 0.5 mmol) was dissolved in 3 mL of anhydrous acetonitrile. Triethylamine (216 μL, 1.5 mmol) and 2-bromoethanol (68 μL, 1 mmol) were added, and the resulting solution was heated at 80 °C for 9 h in an oil bath. The reaction progress was monitored by analytical HPLC (method 1, *t*_R_ = 7.8 min). More 2-bromoethanol (68 μL, 1 mmol) was added to the reaction every 3 h, as necessary, until the reaction reached completion (i.e., all of compound **1** had reacted). The crude product was purified by semipreparative HPLC in small batches (method 3, *t*_R_ = 7.3 min). Isolated yield: 92 mg (54%). The product was characterized by HR-ESI-MS, ^1^H NMR, and ^13^C NMR. HR-ESI-MS (*m*/*z*) calculated for C_19_H_32_N_4_O_3_ [M + H]^+^ 365.2547, found 365.2536. ^1^H NMR (D_2_O, 600 MHz): δ_H_ 7.40–7.30 (m, 5H), 4.40 (s, 2H), 3.87–3.86 (m, 8H), 3.64 (s, 2H), 3.51–3.01 (m, 12H). ^13^C NMR (CD_3_CN, 600 MHz): δ_C_ 173.2, 138.1, 128.5, 127.7, 127.3, 59.4, 56.9, 56.8, 55.9, 51.4, 50.4, 49.1, 43.2.

### 2.5 *N*-Benzyl-2-(4,7-bis(2-oxopropyl)-1,4,7-triazonan-1-yl)acetamide (**4**)

Compound **1** (20 mg, 0.07 mmol) was dissolved in 2.5 mL of anhydrous acetonitrile. Triethylamine (30 μL, 0.2 mmol) and 2-chloroacetone (12 μL, 0.14 mmol) were added, and the resulting solution was heated at 80 °C for 3 h. The reaction progress was monitored by analytical HPLC (method 1, *t*_R_ = 9.4 min). The solvent was removed under reduced pressure, and the crude product was purified by semipreparative HPLC in small batches (method 4, *t*_R_ = 13.6 min). Isolated yield: 26 mg (93%). The product was characterized by HR-ESI-MS, ^1^H NMR, and ^13^C NMR. HR-ESI-MS (*m*/*z*) calculated for C_21_H_32_N_4_O_3_ [M + H]^+^ 389.2547, found 389.2531. ^1^H NMR ((CD_3_)_2_SO, 600 MHz): δ_H_ 8.62 (t, 1H, *J* = 5.7 Hz), 7.35–7.25 (m, 5H), 4.33 (d, 2H, *J* = 5.5 Hz), 3.90 (s, 4H), 3.71 (s, 2H), 3.19–2.75 (m, 12H), 2.05 (s, 6H). ^13^C NMR ((CD_3_)_2_SO, 600 MHz): δ_C_ 206.1, 168.5, 139.4, 128.8, 127.8, 127.4, 63.4, 57.0, 50.2, 49.9, 49.6, 42.6, 27.7.

### 2.6 2,2′-(7-(2-(Benzylamino)-2-oxoethyl)-1,4,7-triazonane-1,4-diyl)diacetic Acid (**5**)

**Scheme 2:**
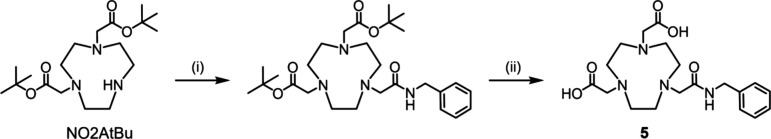
Synthesis of TACN-Based Chelator **5** (i) *N*-Benzyl-2-bromoacetamide (1 equiv), potassium carbonate (1.8 equiv), acetonitrile, RT, 48 h. (ii) Trifluoroacetic acid (850 μL), triisopropylsilane (100 μL), water (50 μL), RT, 6 h. Overall isolated yield: 64%.

The synthesis of chelator **5** is shown in Scheme 2 and was adapted from a previously reported procedure.^25^ Commercially available NO2AtBu (18 mg, 0.05 mmol) was dissolved in 1.5 mL of acetonitrile with potassium carbonate (11.9 mg, 0.09 mmol). *N*-Benzyl-2-bromoacetamide (12 mg, 0.05 mmol) was added, and the reaction was stirred at room temperature for 48 h. The reaction mixture was then filtered, and the solvent was removed under reduced pressure. The crude residue was reconstituted in a mixture of TFA, triisopropylsilane, and water (8.5:1:0.5, 1 mL) and stirred for 6 h at room temperature. The reaction was dried under nitrogen and reconstituted in 3 mL of diethyl ether with <5% methanol, resulting in the formation of a white precipitate. The precipitate was recovered by filtration and washed with dichloromethane. The final product was purified by semipreparative HPLC (*t*_R_ = 8.0 min, method 2). Isolated yield: 35 mg (64%). The product was characterized by HR-ESI-MS, ^1^H NMR, and ^13^C NMR. HR-ESI-MS (*m*/*z*) calculated for C_19_H_28_N_4_O_5_ [M + H]^+^ 393.2133, found 393.2105. ^1^H NMR ((CD_3_)_2_SO, 600 MHz): δ_H_ 8.78 (t, 1H, *J* = 5.4 Hz), 7.34–7.25 (m, 5H), 4.34 (d, 2H, *J* = 5.8 Hz), 3.84 (s, 2H), 3.67 (s, 4H), 3.15–3.00 (m, 12H). ^13^C NMR ((CD_3_)_2_SO, 600 MHz): δ_C_ 171.9, 167.3, 139.2, 128.8, 127.8, 127.4, 57.0, 55.1, 50.9, 49.7, 48.7, 42.7.

### 2.7 Labeling of TACN-Based Chelators with the [^nat^Re(CO)_3_]^+^ Core

The [^nat^Re(CO)_3_(OH_2_)_3_](NO_3_) precursor was synthesized from (NEt_4_)_2_[Re(CO)_3_Br_3_], as previously described.^14^ Details for the synthesis can be found in the Supporting Information. The [^nat^Re(CO)_3_(OH_2_)_3_](NO_3_) precursor was reacted with the TACN-based chelators in a ∼2:1 molar ratio. The optimized reaction conditions for the individual chelators are as follows.

#### 2.7.1 Re-2

Chelator **2** (11.5 mg, 0.029 mmol) and [^nat^Re(CO)_3_(OH_2_)_3_](NO_3_) (23 mg, 0.059 mmol) were combined in 900 μL of phosphate-buffered saline (PBS; 1 mM, pH 9) and heated at 95 °C for 3 h in a thermomixer. The product was purified by semipreparative HPLC (method 1, *t*_R_ = 13.8 min) and characterized by HRMS, IR, ^1^H NMR, ^13^C NMR, and elemental analyses. Isolated yield: 9.0 mg (47%). HR-ESI-MS (*m*/*z*) calculated for C_22_H_29_N_6_O_6_^185^Re^+^ [M]^+^ 661.1780, found 661.1808. IR (solid, cm^–1^): 3312, 3195, 2033, 1905, 1664, 1134. ^1^H NMR (CD_3_CN, 600 MHz): δ_H_ 7.38–7.32 (m, 5H), 7.22 (t, 1H, *J* = 5.6 Hz), 6.47 (s, 2H), 6.00 (s, 2H), 4.40 (d, 2H, *J* = 5.3 Hz), 4.23 (s, 6H), 3.64–3.58 (m, 12H). ^13^C NMR (CD_3_CN, 600 MHz): δ_C_ 194.5, 169.4, 167.3, 138.4, 128.5, 127.5, 127.3, 65.33, 65.30, 57.6, 57.41, 57.39, 42.4. Elemental analysis: anal. calcd. for C_22_H_29_N_6_O_6_Re^+^(TFA)_2_(H_2_O)((CH_3_)_2_CO): C, 35.47; H, 4.21; N, 8.56. Found C, 35.14; H, 3.81; N, 8.42.

#### 2.7.2 Re-3

Chelator **3** (15 mg, 0.041 mmol) and [^nat^Re(CO)_3_(OH_2_)_3_](NO_3_) (25 mg, 0.065 mmol) were combined in 500 μL of aqueous sodium bicarbonate (0.1 M, pH 8.2) and heated at 95 °C for 3 h in a thermomixer. The product was purified by semipreparative HPLC (method 5, *t*_R_ = 7.4 min) and characterized by HRMS, IR, ^1^H NMR, ^13^C NMR, and elemental analyses. Isolated yield: 9.6 mg (37%). HR-ESI-MS (*m*/*z*) calculated for C_22_H_31_N_4_O_6_^185^Re^+^ [M]^+^ 635.1875, found 635.1899. IR (solid, cm^–1^): 3100–3600, 2917, 2850, 2029, 1903, 1671, 1176, 1128. ^1^H NMR (CD_3_CN, 600 MHz): δ_H_ 7.39–7.29 (m, 5H), 7.22 (s, 1H), 4.67 (t, 1H, *J* = 4.7 Hz), 4.40 (d, 2H, *J* = 6.0 Hz), 4.25 (m, 2H), 3.86–3.89 (m, 4H), 3.69–3.38 (m, 12H), 3.32–3.26 (m, 4H). ^13^C NMR (CD_3_CN, 600 MHz): δ_C_ 195.5, 167.4, 138.5, 128.5, 127.5, 127.2, 68.1, 66.2, 58.5, 57.6, 57.0, 56.9, 42.5. Elemental analysis: anal. calcd. for C_22_H_31_N_4_O_6_Re^+^(TFA)_2_: C, 36.20; H, 3.97; N, 6.49. Found C, 35.96; H, 3.85; N, 6.20.

#### 2.7.3 Re-4

Chelator **4** (1 mg, 2.6 μmol) and [^nat^Re(CO)_3_(OH_2_)_3_](NO_3_) (2 mg, 5.2 μmol) were combined in 500 μL of 0.2 M MES (2-(*N*-morpholino)ethanesulfonic acid) buffer pH 4–6, 0.1 M sodium acetate pH 4–5, 0.1 M sodium bicarbonate pH 7–9, 0.1 M sodium carbonate pH 10–12, 1 mM PBS pH 7–12, or organic solvent (methanol or dimethyl sulfoxide) and heated at 95 °C for 3 h in a thermomixer. Labeling was unsuccessful under all tested conditions.

#### 2.7.4 Re-5

Chelator **5** (10 mg, 0.025 mmol) and [^nat^Re(CO)_3_(OH_2_)_3_](NO_3_) (20 mg, 0.052 mmol) were combined in 500 μL of MES buffer (0.2 M, pH 5) and heated at 95 °C for 1 h in a thermomixer. The product was purified by semipreparative HPLC (method 5, *t*_R_ = 7.8 min) and characterized by HRMS, IR, ^1^H NMR, ^13^C NMR, and elemental analyses. Isolated yield: 8.7 mg (53%). HR-ESI-MS (*m*/*z*) calculated for C_22_H_27_N_4_O_8_^185^Re^+^ [M]^+^ 663.1460, found 663.1445. IR (solid, cm^–1^): 2032, 1903, 1725, 1660. ^1^H NMR (CD_3_CN, 600 MHz): δ_H_ 7.45–7.28 (m, 5H), 4.40 (d, 2H, *J* = 5.6 Hz), 4.34 (s, 4H), 4.24 (s, 2H), 3.68–3.51 (m, 12H). ^13^C NMR (CD_3_CN, 600 MHz): δ_C_ 194.3, 169.4, 167.4, 138.4, 128.5, 127.5, 127.3, 66.1, 64.9, 57.6, 57.3, 42.6. Elemental analysis: anal. calcd. for C_22_H_27_N_4_O_8_Re^+^(TFA)_2_: C, 35.06; H, 3.39; N, 6.29. Found C, 35.29; H, 3.51; N, 6.53.

### 2.8 Radiolabeling of TACN-Based Chelators with the [^99m^Tc][Tc(CO)_3_]^+^ Core

High specific activity [^99m^Tc]TcO_4_^–^, eluted in saline from a ^99^Mo/^99m^Tc generator, was used to synthesize the [^99m^Tc][Tc(CO)_3_(OH_2_)_3_]^+^ precursor, as previously reported.^13^ The optimized reaction conditions for the individual chelators are as follows.

#### 2.8.1 [^99m^Tc]Tc-2

Chelator **2** (35 μg, 0.09 μmol) and [^99m^Tc][Tc(CO)_3_(OH_2_)_3_]^+^ (3.7–55.5 MBq, 0.1–1.5 mCi) were combined in 300 μL of sodium bicarbonate (0.1 M, pH 7) and heated at 95 °C for 1 h.

#### 2.8.2 [^99m^Tc]Tc-3

Chelator **3** (33 μg, 0.09 μmol) and [^99m^Tc][Tc(CO)_3_(OH_2_)_3_]^+^ (3.7–55.5 MBq, 0.1–1.5 mCi) were combined in 300 μL of sodium bicarbonate (0.1 M, pH 9) and heated at 95 °C for 1 h.

#### 2.8.3 [^99m^Tc]Tc-4

Chelator **4** was not successfully reacted with [^99m^Tc][Tc(CO)_3_(OH_2_)_3_]^+^ under all tested conditions.

#### 2.8.4 [^99m^Tc]Tc-5

Chelator **5** (35 μg, 0.09 μmol) and [^99m^Tc][Tc(CO)_3_(OH_2_)_3_]^+^ (3.7–55.5 MBq, 0.1–1.5 mCi) were combined in 300 μL of MES buffer (0.2 M, pH 5) and heated at 95 °C for 1 h.

Reaction progress was monitored by radio-HPLC, and the formation of colloidal ^99m^TcO_2_ was monitored by radio-TLC (colloid *R*_f_ = 0; other solution components near *R*_f_ = 1, Supporting Information Figures S19–S21). All ^99m^Tc-labeled complexes were characterized by HPLC coinjection with the fully characterized ^nat^Re-labeled analogues.

### 2.9 Radiolabeling of TACN-Based Chelators with the [^186^Re][Re(CO)_3_]^+^ Core

Low specific activity [^186^Re]ReO_4_^–^, produced by neutron irradiation of an enriched ^185^Re target, was used to synthesize the [^186^Re][Re(CO)_3_(OH_2_)_3_]^+^ precursor as previously reported.^16^ The optimized reaction conditions for the individual chelators are as follows.

#### 2.9.1 [^186^Re]Re-2

Chelator **2** (195 μg, 0.5 μmol) and [^186^Re][Re(CO)_3_(OH_2_)_3_]^+^ (0.7–7.4 MBq, 20–200 μCi) were combined in 500 μL of sodium bicarbonate (0.1 M, pH 8) and heated at 95 °C for 1 h.

#### 2.9.2 [^186^Re]Re-3

Chelator **3** (182 μg, 0.5 μmol) and [^186^Re][Re(CO)_3_(OH_2_)_3_]^+^ (0.7–1.9 MBq, 20–50 μCi) were combined in 500 μL of sodium bicarbonate (0.1 M, pH 9) and heated at 95 °C for 1 h.

#### 2.9.3 [^186^Re]Re-4

Chelator **4** was not successfully reacted with [^186^Re][Re(CO)_3_(OH_2_)_3_]^+^.

#### 2.9.4 [^186^Re]Re-5

Chelator **5** (59 μg, 0.15 μmol) and [^186^Re][Re(CO)_3_(OH_2_)_3_]^+^ (0.7–37 MBq, 0.02–1 mCi) were combined in 500 μL of MES (0.2 M, pH 5) and heated at 95 °C for 1 h.

Reaction progress was monitored by radio-HPLC, and the formation of colloidal ^186^ReO_2_ was monitored by radio-TLC (colloid *R*_f_ = 0; other solution components near *R*_f_ = 1, Supporting Information Figures S22–S24). All ^186^Re-labeled complexes were characterized by HPLC coinjection with the fully characterized ^nat^Re-labeled analogues.

### 2.10 *In Vitro* Stability and logD_7.4_ Studies

The crude radiocomplexes were isolated by radio-HPLC, and the HPLC eluent was evaporated under a nitrogen gas stream until the volume was reduced to 50–100 μL. The purified radiocomplex was then added to a solution of PBS buffer (350–400 μL, 1 mM, pH 7.4), l-cysteine (1 mM in 350–400 μL PBS), l-histidine (1 mM in 350–400 μL PBS), or rat serum (400–450 μL, Innovative grade US Origin Sprague–Dawley Rat Serum, Innovative Research, Novi, Michigan). Ascorbic acid (50 μL, 1 mg/mL in PBS) was added as a radioprotectant, and the solutions were incubated at 37 °C. Aliquots of each solution (PBS, l-cysteine, and l-histidine) were taken at 24 h, and the stability of the radiocomplexes was evaluated by radio-HPLC and radio-TLC. Aliquots (100–500 μL) of rat serum were taken at 24 h and combined with a 4× volume of acetonitrile to precipitate the serum proteins. The resulting mixture was vortexed (5 min) and centrifuged (10 min, 1900 × *g*), and the supernatant was carefully removed. The precipitated proteins were washed with an additional 100–500 μL of acetonitrile, vortexed (5 min), and centrifuged (10 min, 1900 × *g*), and the supernatant was carefully removed. The combined supernatants were used to determine radiocomplex stability by radio-HPLC and radio-TLC analyses. The supernatant and pellet activities were used to determine the percent protein binding of the radiocomplex.

The distribution coefficients were determined with octanol/water partitioning. The HPLC-purified radiocomplexes were diluted to 500 μL in PBS (1 mM, pH 7.4) and added to a mixture of 5 mL of octanol and 4.5 mL of PBS. The resulting solutions were vortexed (3 min) and centrifuged (10 min, 1900 × *g*). The octanol and PBS layers were separated and aliquoted into four 1 mL fractions. The activity in each fraction was counted on either an HPGe detector (^186^Re-complexes) or NaI(Tl) well detector (^99m^Tc-complexes). Each logD_7.4_ value was calculated by taking the log of the average counts in the organic layer divided by the average counts in the aqueous layer. This experiment was conducted in triplicate for each radiocomplex. Each reported logD_7.4_ value represents an average of the three experiments.

### 2.11 Single-Crystal X-ray Diffraction Analysis

Attempts to grow X-ray quality crystals of ^**nat**^**Re-2**/**3**/**5** were unsuccessful. However, X-ray quality crystals of a related [^nat^Re(CO)_3_]^+^-labeled TACN-based chelator bearing one *N*-benzyl-acetamide arm and two ethyl ester arms (the synthesis of which was previously reported^16^) were successfully grown by the slow evaporation of a 50% ethanol in water solution of the compound over the course of 3 weeks. Single-crystal X-ray diffraction (SCXRD) data were measured on a Bruker D8 Venture diffractometer with a Photon II CMOS area detector using Mo Kα radiation from a microfocus source (Bruker AXS, Madison, Wisconsin, USA). The crystal was cooled to 173.0 K under a cold stream of N_2_ using a Cryostream 800 cryostat (Oxford Cryosystems, Oxford, UK). A hemisphere of unique data was collected using strategies of scans about the phi and omega axes. The Apex4 software suite was used for data collection, unit cell determination, data reduction, absorption correction and scaling, and space group determination.^26^

The structure was solved in the monoclinic space group P2_1_/*n* by direct methods, as implemented in SHELXS^27^ and refined by full matrix least-squares refinement using SHELXL v.2017.^28^ Olex2 was used as an interface for structure visualization and model building.^29^ Non-hydrogen atoms were located from the difference map and refined anisotropically. Carbon atoms in the heterocyclic core were found to be disordered and refined over two positions located from the difference map with their occupancies set to values that gave similar isotropic thermal parameters (30%/70% for one group and 40%/60% for the other). The amide N–H hydrogen atom was located from the difference map, and its coordinates were refined with the N–H distance restrained to 0.92(1) Å. All other hydrogen atoms were placed in calculated positions, and their coordinates and thermal parameters were constrained to ride on the carrier atoms. The final model has a significant difference map peak near the Re atom, which has the same *y* coordinates as the Re atom generated by a glide operation and is attributed to a packing fault where chains of molecules related by the glide operation substitute for each other. The [CF_3_COO]^−^ ion shows evidence for positional disorder of the entire molecule, but the minor part failed to refine to a realistic geometry consistent with hydrogen bonding to the amide. Refining this group anisotropically at full occupancy gave the best model but with some anomalously short C–O and C–F distances likely due to the imprecision of these atomic positions. Crystal data, including selected bond lengths and angles, can be found in the Supporting Information.

## 3 Results and Discussion

### 3.1 Synthesis of Modified TACN Chelators

The synthesis of the modified TACN chelators is shown in Schemes 1 and 2. Chelators **2**–**4** were synthesized from commercially available TACN following two synthetic methods. In the first method, TACN was reacted directly with *N*-benzyl-2-bromoacetamide in a 1:1 molar ratio. This reaction resulted in the formation of three products in a 6:3:1 ratio: the desired monosubstituted *N-*benzyl acetamide product (**1**), a disubstituted *N-*benzyl acetamide chelator, and a trisubstituted chelator. The three products were easily separated by HPLC isolation, resulting in a 40% isolated yield for the desired product, **1**. Given the multiple side products formed that affected the overall yield of the desired product, an alternative synthetic method was used (Scheme S1 in the Supporting Information). This method began with the reaction of TACN with *N,N*-dimethylformamide dimethyl acetal to form TACN-orthoamide in near quantitative yields. The orthoamide intermediate allowed for preferential formation of the monosubstituted product upon reaction with *N*-benzyl-2-bromoacetamide,^30^ which was then reacted with sodium hydroxide to convert the orthoamide back to an unrestricted TACN ring and form chelator **1**. The overall isolated yield for the orthoamide approach was 50–60%, with loss in yield attributed to incomplete addition of the *N-*benzyl acetamide arm, minor formation of the di- and trisubstituted products (<10%), and/or hydrolysis of the amide bond by the sodium hydroxide (as confirmed by LC-ESI-MS analysis).

Chelators **2**–**4** were formed by heating chelator **1** with 2-bromoacetamide, 2-bromoethanol, or 2-chloroacetone, respectively, in acetonitrile with triethylamine as the base at 80 °C for 3–9 h. The progress of these reactions was monitored by HPLC and LC-ESI-MS analyses, revealing a very clean synthesis with minimal byproduct formation. The chelators were purified via semipreparative HPLC with isolated yields between 50 and 90%. These reactions were easily scaled up from 5 to 10 mg to 100–200 mg with minimal impact on yield. The purified chelators were isolated as yellow or colorless oils and were fully characterized prior to (radio)labeling studies. Chelators **2** (diamide) and **3** (dialcohol) were soluble in water, methanol, acetonitrile, and dimethyl sulfoxide and remained stable when stored at 4 °C for several weeks. Chelator **4** (diketone) was soluble in dimethyl sulfoxide and remained stable at 4 °C for several weeks.

Chelator **5** (diacid) was used as a comparator for these experiments, since high (radio)labeling yields and excellent stability of NOTA derivative complexes with the [M(CO)_3_]^+^ cores is well established in the literature.^15,16,20^ Chelator **5** was synthesized in high yield from commercially available NO2AtBu following a literature procedure (Scheme 2).^25^ Prior to (radio)labeling studies, the chelator was isolated by HPLC as a colorless oil and fully characterized. The chelator was soluble in water, methanol, acetonitrile, and dimethyl sulfoxide and remained stable at room temperature for several months.

### 3.2 Chelator Labeling with the [^nat^Re(CO)_3_]^+^ Core

Since the synthesis of radiometal complexes is performed at extremely small scales (nmol scale or lower in these studies), traditional characterization techniques cannot be used to confirm their chemical structures. Thus, chelators **2**–**5** were labeled with the [^nat^Re(CO)_3_]^+^ core, and the resulting products were fully characterized for subsequent use as nonradioactive metal complex standards for comparison against the radiocomplexes via HPLC coinjection. These ^nat^Re-labeled complexes served as standards for both the ^186^Re- and ^99m^Tc-labeled complexes because there are no nonradioactive isotopes of technetium. Rhenium and technetium belong to the same group of the periodic table, resulting in them having similar chemical and physical properties. As such, rhenium and technetium were expected to coordinate to the TACN chelators in a matching manner.^15,16^

Chelator **2** (diamide) was reacted with the [^nat^Re(CO)_3_(OH_2_)_3_]^+^ core in PBS buffer (pH 7–9) at 95 °C for 3 h in a thermomixer, with pH 9 resulting in the highest yields. The reaction progress was monitored by HPLC (*t*_R_ = 11.0 min, method 1). After 3 h, only ∼50% of the chelator had been successfully labeled (by HPLC), leading to an isolated yield of 40%. Due to decomposition of the [^nat^Re(CO)_3_(OH_2_)_3_]^+^ precursor, further heating was not expected to increase the labeling yield. The reaction was also tested in MES buffer (0.2 M, pH 3–6), PBS (1 mM, pH 3–7 and 10–12), sodium bicarbonate (0.1 M, pH 7–9), and sodium carbonate (0.1 M, pH 10–12). The labeling was unsuccessful at a pH less than 7 or greater than 9, regardless of the buffer. In sodium bicarbonate, two products were formed: the desired ^**nat**^**Re-2** product and a byproduct with a mass to charge ratio corresponding to 1 Da greater than the ^**nat**^**Re-2** complex (LC-ESI-MS, *t*_R_ = 18.5 min, method 6). The formation of this byproduct was attributed to the base hydrolysis of the amide group, converting it to a carboxylic acid.^31^ The formation of the hydrolysis product was limited by performing the labeling reaction in sodium bicarbonate under microwave heating at 95 °C for 30 min. Microwave heating had the added benefit of a shorter reaction time while resulting in a yield similar to that achieved from 3 h of conventional heating in PBS buffer (∼40%). Performing the microwave reaction in PBS did not significantly increase the yield.

Chelator **3** (dialcohol) was reacted with [^nat^Re(CO)_3_(OH_2_)_3_]^+^ in sodium bicarbonate (0.1 M, pH 7–9) at 95 °C for 3 h in a thermomixer. The yield (by HPLC) was near quantitative throughout that pH range with minimal byproduct formation observed. The labeling reaction was successful at pH 10–12 in sodium carbonate (0.1 M); however, these reaction conditions were not used for labeling of more than 1 mg of chelator due to the instability of [^nat^Re(CO)_3_(OH_2_)_3_]^+^ at very high pH.^32^ Sodium bicarbonate was ultimately chosen as the optimal buffer.

Chelator **4** (diketone) proved more difficult to label. Chelator **4** was reacted with [^nat^Re(CO)_3_(OH_2_)_3_]^+^ in sodium acetate (0.1 M, pH 4–5), MES buffer (0.2 M, pH 4–6), sodium bicarbonate (0.1 M, pH 7–9), PBS (1 mM, pH 7–12), sodium carbonate (0.1 M, pH 10–12), and organic solvent (30% methanol in water or dimethyl sulfoxide) with heating at 95 °C for 3 h. Under all tested reaction conditions, successful labeling was not achieved. Alternative routes to synthesize ^**nat**^**Re-4** from ^**nat**^**Re-1** (nonfunctionalized) or ^**nat**^**Re-5** (diacid) were explored but deemed impractical due to long reaction times and the excessive reagents required to achieve the chemical conversions.

Previous labeling investigations of TACN-based chelators with the [M(CO)_3_]^+^ core found an association between chelators with ionizable pendent group(s) and increased labeling efficiency.^16^ Successful labeling was only demonstrated with chelators that were not sterically hindered (i.e., had no pendent arms, **1**) or that bore pendent arms that were ionized under the labeling conditions (e.g., the diacid, **5**, and mixed acid/ester pendent arms). The TACN-based chelators bearing exclusively ester pendent arms were not successfully labeled and had to be indirectly synthesized by esterification of the [^nat^Re(CO)_3_]^+^-labeled diacid chelator (^**nat**^**Re-5**). The inability to directly label the diester chelators was postulated to be due, in part, to the lack of an electrostatic attraction between the pendent arms and the metal center.

**Figure 1:**
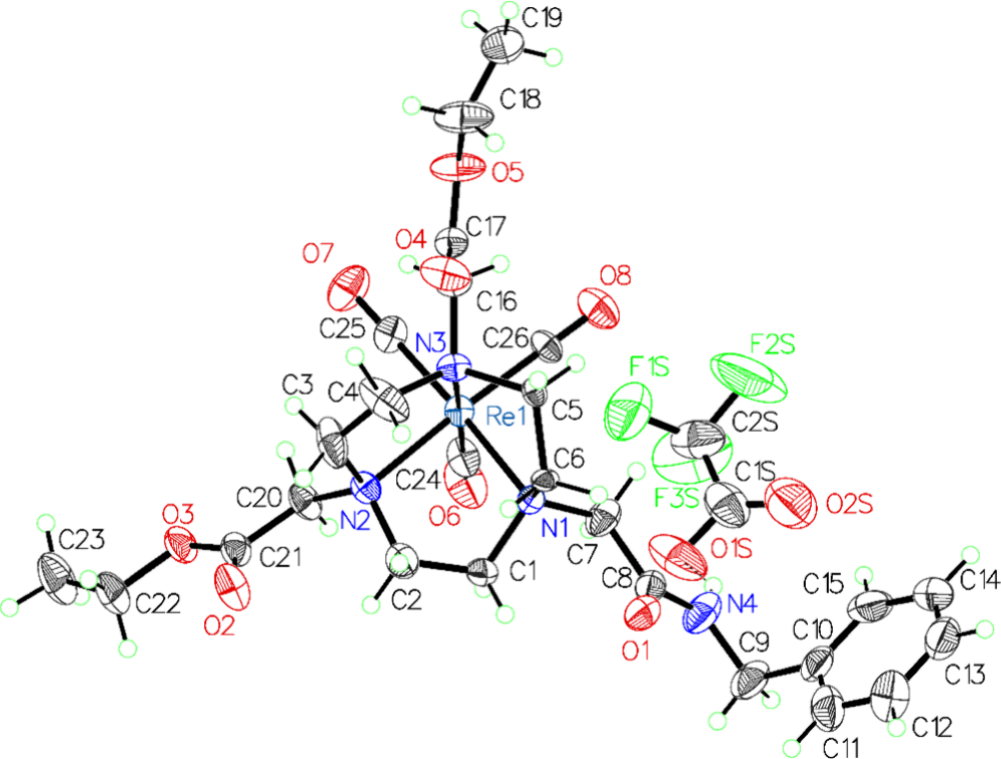
Crystal structure of the [^nat^Re(CO)_3_]^+^-labeled TACN-based chelator bearing one *N*-benzyl-acetamide arm and two ethyl ester arms. The crystal structure demonstrates the distorted octahedral coordination of the rhenium metal center with three *fac*-coordinated carbon monoxide ligands and the three nitrogens in the TACN backbone. The pendent arms do not participate in binding to the metal center in the final metal complex. The pendent amide arm is hydrogen bonded with a trifluoroacetate anion. Labeled 50% probability ellipsoid plot of asymmetric unit; disorder omitted for clarity.

Consistent with those findings, chelator **4** (diketone), which bears nonpolar pendent arms that do not ionize under the labeling conditions, was not successfully labeled with the [^nat^Re(CO)_3_]^+^ core, even despite the smaller size (steric bulk) of its pendent arms as compared with the previously studied diester derivatives. The pendent arms on the modified TACN chelators clearly impact the efficiency and the extent of labeling, yet they do not participate in coordination of the metal center in the final complex. The latter is evidenced by both the X-ray crystal structure shown in Figure 1 for the [^nat^Re(CO)_3_]^+^-labeled diethyl ester TACN derivative chelator as well as the previously reported X-ray crystal structures of the [^nat^Re(CO)_3_]^+^- and [^99g^Tc(CO)_3_]^+^-labeled NOTA complexes reported by Braband et al.^23^

The amide and alcohol pendent arms of chelators **2** and **3**, respectively, are also not ionized under the labeling conditions; however, their polar functional groups bear partial negative charges, which appear to be sufficient for electrostatic attraction of the positively charged metal center to facilitate coordination. Such an attraction could occur directly between the partial negative terminal polar group and the positive metal center. Additionally, as supported by the crystal structure in Figure 1, indirect interactions may also occur, wherein polar pendent groups can form strong hydrogen bonds with anions in solution (e.g., trifluoroacetate or bicarbonate anions). The hydrogen bonded, noncoordinating anion thereby creates an anionic region around the chelator that could aid in electrostatically attracting the positively charged metal center to facilitate its coordination with the TACN backbone. The noncoordinating anions may also be positioned near enough to the metal center where they may engage in stabilizing outer sphere Coulombic interactions. As also depicted in the crystal structure, nonpolar pendent arms (e.g., ester or ketone) do not form hydrogen bonds with anions in solution, consistent with diminished attraction of the metal center.

As previously reported, chelator **5** (diacid) was reacted with [^nat^Re(CO)_3_(OH_2_)_3_]^+^ in MES buffer (0.2 M, pH 3–5) at 95 °C for 1 h in a thermomixer. No significant difference in yield was observed across that pH range.^16^ The yield (by HPLC) was near quantitative with no formation of byproducts observed.

All ^**nat**^**Re-X** complexes were soluble in water and acetonitrile and stable for several months at room temperature. The ^**nat**^**Re-X** complexes were isolated as hygroscopic white powders and characterized by HR-ESI-MS, ^1^H NMR, ^13^C NMR, IR, and elemental analyses. While attempts to grow X-ray quality crystals of ^**nat**^**Re-2**/**3**/**5** have thus far been unsuccessful, the ^**nat**^**Re-X** complexes reported in this work are expected to have similar coordination properties to the [^nat^Re(CO)_3_]^+^-labeled diethyl ester complex shown in Figure 1. HR-ESI-MS analyses confirmed the anticipated mass to charge ratio and isotopic distribution for each of the ^**nat**^**Re-X** complexes. ^1^H NMR analyses confirmed the anticipated structures and provided evidence that the pendent arms did not coordinate to the metal center. For example, coordination of the [^nat^Re(CO)_3_]^+^ core results in a distinctive downfield shift for the hydrogen atoms of the TACN ring (3.1 to 3.6 ppm for **Re-2** and 3.4 to 3.6 ppm for **Re-3**). The metal coordination sphere is filled by the three TACN backbone nitrogens and the three carbon monoxide ligands. The presence of the carbon monoxide ligands was confirmed with both ^13^C NMR spectra (chemical shifts at 195 ppm) and IR spectra (∼2030 cm^–1^ symmetrical stretching and ∼1900 cm^–1^ overlapping antisymmetric stretching^14^). Elemental analysis confirmed the anticipated elemental composition of the ^**nat**^**Re-X** complexes as TFA salts.

### 3.3 Chelator Radiolabeling with the [^99m^Tc][Tc(CO)_3_]^+^ and [^186^Re][Re(CO)_3_]^+^ Cores

**Figure 2:**
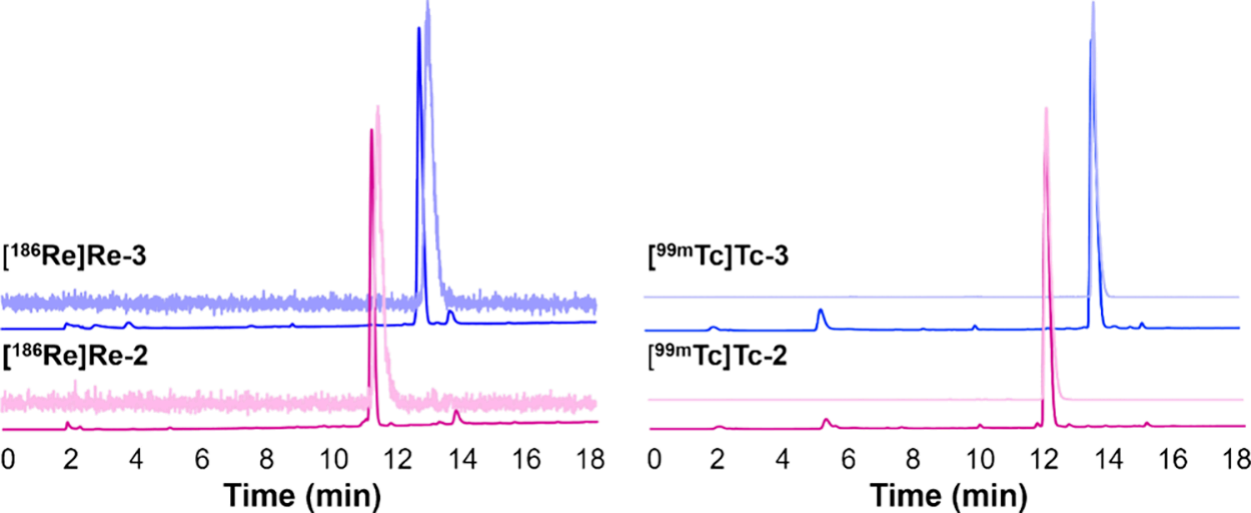
HPLC coinjections (method 1) of the radiocomplexes (lighter colors; NaI(Tl) detector) with their fully characterized ^**nat**^**Re-X** counterparts (darker colors; UV detector, 254 nm). Differences in retention times between the **[**^**186**^**Re]Re-X** and **[**^**99m**^**Tc]Tc-X** complexes are attributed to different HPLCs being used for analysis.

For radiolabeling studies, high specific activity [^99m^Tc]TcO_4_^–^ was eluted from a ^99^Mo/^99m^Tc generator in saline. The [^99m^Tc]TcO_4_^–^ was converted to the [^99m^Tc][Tc(CO)_3_(OH_2_)_3_]^+^ precursor following an established literature procedure.^13^ The pH of the precursor (∼10 upon successful synthesis) was not adjusted, except for radiolabeling of chelator **5**, in which case 6 M HCl was added to reduce the pH to ∼5. After combining the precursor, buffer, and chelator, the reaction pH for labeling of chelators **2** and **3** was adjusted, as needed, with 1 M HCl prior to heating. The conditions for radiolabeling were optimized for each chelator in terms of buffer, buffer pH, temperature, and ligand concentration. Characterization of the radiocomplexes was conducted by HPLC coinjection with the ^**nat**^**Re-X** counterparts (Figure 2; coinjection data for **M-5** were reported previously^16^).

Radiochemical yields (RCYs) for the radiocomplexes, obtained by HPLC peak area integration coupled with radio-TLC analysis of colloid formation, are listed in Table 1. Chelators **2** and **3** were reacted with the [^99m^Tc][Tc(CO)_3_(OH_2_)_3_]^+^ precursor in sodium bicarbonate (0.1 M, pH 7–9) at 95 °C for 1 h, with pH 7 giving the highest RCY of 46 ± 8% for **[**^**99m**^**Tc]Tc-2** and pH 9 giving the highest RCY of 35 ± 6% for **[**^**99m**^**Tc]Tc-3**. The RCY of **[**^**99m**^**Tc]Tc-3** was increased to ∼45% using the same reaction conditions, except for heating the reaction in the microwave at 95 °C for 20 min. As observed with the ^nat^Re-labeling reactions, neutral to basic conditions were required for the labeling to occur. The reactions were unsuccessful at a pH of less than 7 (in MES buffer or PBS) or greater than 10 (in 0.1 M sodium carbonate). Increasing the reaction time had no effect on yield, since the [^99m^Tc][Tc(CO)_3_(OH_2_)_3_]^+^ precursor had either reacted with the chelator or oxidized to [^99m^Tc]TcO_4_^–^ (as evidenced by HPLC) in 1 h. No colloidal [^99m^Tc]TcO_2_, however, was observed by radio-TLC during the labeling reactions.

**Table 1:** Radiochemical Yields, logD_7.4_ Values, Percent Stability, and Percent Protein Binding for the Radiolabeled Chelators^a^

**M = [**^**99m**^**Tc]Tc**	M-2 (diamide)	M-3 (dialcohol)	M-5 (diacid)^c^
**RCY (%)**	46 ± 8	35 ± 6	96 ± 1
**logD**_**7.4**_	–1.15 ± 0.01	–0.27 ± 0.01	–2.2 ± 0.3
**stability in PBS**^b^**(%)**	100 ± 0	100 ± 0	100 ± 0
**stability in**l**-cysteine**^b^**(%)**	100 ± 0	100 ± 0	100 ± 0
**stability in**l**-histidine**^b^**(%)**	100 ± 0	100 ± 0	100 ± 0
**rat serum protein binding**^b^**(%)**	1 ± 1	3 ± 1	8 ± 2

**M = [**^**186**^**Re]Re**	M-2 (diamide)	M-3 (dialcohol)	M-5 (diacid)^c^
**RCY (%)**	8 ± 1	32 ± 3	96 ± 1
**logD**_**7.4**_	–1.04 ± 0.02	–0.19 ± 0.06	–1.9 ± 0.2
**stability in PBS**^b^**(%)**	100 ± 0	100 ± 0	100 ± 0
**stability in**l**-cysteine**^b^**(%)**	100 ± 0	100 ± 0	100 ± 0
**stability in**l**-histidine**^b^**(%)**	100 ± 0	100 ± 0	100 ± 0
**rat serum protein binding**^b^**(%)**	3 ± 1	3 ± 1	5 ± 4

a Values are reported as mean ± SD (*n* = 3).

b Following incubation at 37 °C for 24 h.

c Reproduced from ref (16). Copyright 2023 American Chemical Society.

A high ligand concentration of 1 mM was required for radiolabeling to achieve the highest RCY. Although this concentration is higher than typically used for radiopharmaceutical development, it is consistent with previously reported [M(CO)_3_]^+^-labeled TACN derivative complexes.^16,33^ Due to the lipophilic character of the [M(CO)_3_]^+^ core, radiolabeling of the TACN-based chelators reduces the overall hydrophilicity of the complexes and results in a significant shift to a later HPLC retention time for the labeled complex. This conveniently allows for a relatively simple separation of the radiolabeled product from the excess unlabeled chelator, with a corresponding increase in apparent molar activity of the radiocomplex. However, the need for a postradiosynthesis purification step prior to dose formulation is not ideal and may limit the potential for application of such radiopharmaceuticals in the clinical setting. The high temperature of 95 °C used to achieve the maximum RCY is also a disadvantage, for example by precluding the use of temperature-sensitive biological targeting vectors (e.g., antibodies) or by requiring labeling at lower temperatures with longer reaction times and concomitant lower RCY.

The yield of **[**^**99m**^**Tc]Tc-2** was ∼20% higher in sodium bicarbonate than in PBS buffer, which differed from the results obtained for ^**nat**^**Re-2**. Formation of the amide hydrolysis product was also seen with **[**^**99m**^**Tc]Tc-2**; however, the yield of this byproduct was negligible (<10%) compared to the yield of the **[**^**99m**^**Tc]Tc-2** product. **[**^**99m**^**Tc]Tc-3** was labeled under the same conditions used for ^**nat**^**Re-3**, and similar to ^**nat**^**Re-3**, minimal byproduct formation was observed during radiolabeling. Loss of yield was attributed to oxidation of the [^99m^Tc][Tc(CO)_3_(OH_2_)_3_]^+^ precursor to [^99m^Tc]TcO_4_^–^ for both the **[**^**99m**^**Tc]Tc-2** and **[**^**99m**^**Tc]Tc-3** complexes. Both complexes were successfully isolated by radio-HPLC for characterization and for conducting *in vitro* stability and logD_7.4_ studies.

Chelator **4** was not successfully reacted with the [^99m^Tc][Tc(CO)_3_(OH_2_)_3_]^+^ precursor. Alternative syntheses for **[**^**99m**^**Tc]Tc-4** from **[**^**99m**^**Tc]Tc-5** were investigated but deemed impractical due to the long reaction times required for the acid to ketone conversion, which would exceed several half-lives of ^99m^Tc.

As reported previously,^16^ chelator **5** was reacted with [^99m^Tc][Tc(CO)_3_(OH_2_)_3_]^+^ in MES buffer (0.2 M, pH 5) at 95 °C for 30 min, resulting in near quantitative yields by radio-HPLC. A ligand concentration of 0.3 mM was typically used for radiolabeling but could be decreased to 0.1 mM without significant impact on RCY. No colloidal [^99m^Tc]TcO_2_ formation was observed by radio-TLC.

Low specific activity [^186^Re]ReO_4_^–^ in saline, produced by neutron irradiation of enriched [^185^Re]Al(ReO_4_)_3_ targets, was used for radiolabeling studies. Due to the slower reaction kinetics and higher reduction potential of rhenium compared to technetium, a decrease in RCY was expected for the **[**^**186**^**Re]Re-X** complexes. Indeed, chelators **2** and **3** proved more difficult to label with the [^186^Re][Re(CO)_3_(OH_2_)_3_]^+^ precursor. Prior to labeling studies, sodium hydroxide or sodium bicarbonate was added to the precursor to raise the pH to ∼8–9. Chelator **2** (diamide) and [^186^Re][Re(CO)_3_(OH_2_)_3_]^+^ were combined in sodium bicarbonate (0.1 M, pH 8) and heated at 95 °C for 1 h, resulting in a maximum RCY of 8 ± 1%, for which a ligand concentration of 1 mM was required. Loss of yield was attributed to both the oxidation of the metal to the permetallate form (no colloidal [^186^Re]ReO_2_ was observed) along with the simultaneous formation of the labeled amide hydrolysis product in a ∼1:1 ratio. After ∼1 h of reaction time at 95 °C in the presence of base, the [^186^Re][Re(CO)_3_(OH_2_)_3_]^+^ precursor was completely oxidized to the permetallate form and thus no longer able to react with the chelator. To isolate sufficient activity for *in vitro* stability, logD_7.4_, and characterization studies of the radiocomplex, the radiolabeling reaction was run for 3 h, with additional aliquots of the [^186^Re][Re(CO)_3_(OH_2_)_3_]^+^ precursor (7.4 MBq, 200 μCi) added every 30 min, in an attempt to extend the presence of the [^186^Re][Re(CO)_3_(OH_2_)_3_]^+^ precursor in solution for reaction with the chelator. Using this approach, the radiolabeling was successfully scaled up to activities of 44 MBq (1.2 mCi).

Chelator **3** (dialcohol) and [^186^Re][Re(CO)_3_(OH_2_)_3_]^+^ were combined in sodium bicarbonate (0.1 M, pH 9) and heated at 95 °C for 1 h, resulting in a maximum RCY of 32 ± 3%. A ligand concentration of 1 mM was required to achieve this yield. The RCY was highly dependent on the activity concentration. Attempts to scale up the labeling reaction from ∼1.9 MBq (50 μCi) to ∼7.4 MBq (200 μCi), by increasing the volume of precursor without changing the total volume of the reaction, led to formation of several byproducts with similar retention times (±0.4 min) to the desired product by radio-HPLC analysis. To minimize the formation of these byproducts, the reactions were diluted in sodium bicarbonate (0.1 M, pH 9) so that the volume of [^186^Re][Re(CO)_3_(OH_2_)_3_]^+^ added did not exceed 10% of the total volume. This byproduct formation was not observed with either the ^**nat**^**Re-3** complex or the **[**^**99m**^**Tc]Tc-3** complex. No colloidal [^186^Re]ReO_2_ was observed by radio-TLC. All loss of yield was attributed to oxidation of the metal, with minimal byproduct formation under the optimized conditions.

Due to the inability to label chelator **4** (diketone) with both the [^nat^Re(CO)_3_]^+^ and [^99m^Tc][Tc(CO)_3_]^+^ cores, reaction of chelator **4** with the [^186^Re][Re(CO)_3_(OH_2_)_3_]^+^ precursor was not attempted.

Chelator **5** (diacid), as expected, reacted in high yields with the [^186^Re][Re(CO)_3_(OH_2_)_3_]^+^ precursor in MES buffer (0.2 M, pH 5) at 95 °C for 1 h.^16^ The RCY was near quantitative, and the reaction was easily scaled to higher activity concentrations. Ligand concentrations of 0.1 to 0.3 mM were used without a significant impact on the RCY. No colloidal [^186^Re]ReO_2_ was observed by radio-TLC.

### 3.4 *In Vitro* Stability and logD_7.4_ Values of the Radiocomplexes

The *in vitro* stability of the HPLC-purified radiocomplexes was tested in solutions of PBS, l-cysteine, and l-histidine as well as in rat serum at 37 °C through 24 h. The stability for the standard **M-5** complexes was reported previously.^16^ In short, both the **[**^**99m**^**Tc]Tc-5** and **[**^**186**^**Re]Re-5** complexes remained completely intact under all tested conditions at 37 °C through 24 h, as monitored by radio-HPLC and radio-TLC.

**Figure 3:**
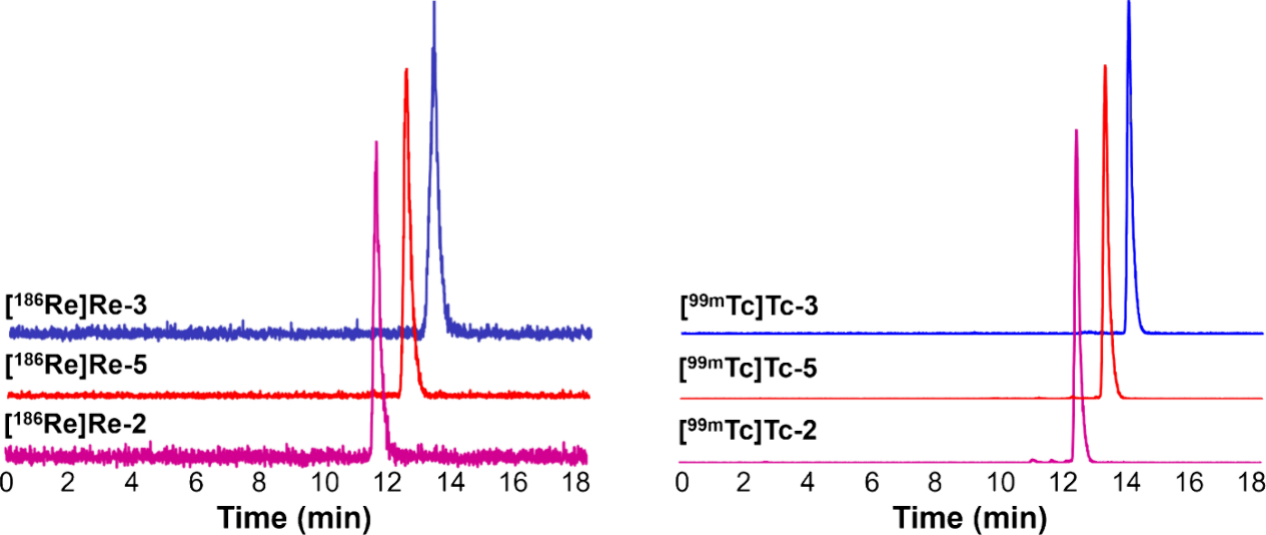
HPLC analysis (method 1; NaI(Tl) detector) of radiocomplex stability after 24 h in rat serum at 37 °C.

Similarly, the **[**^**99m**^**Tc]Tc-2**/**3** and **[**^**186**^**Re]Re-2**/**3** complexes remained completely stable in PBS, l-cysteine, and l-histidine challenge experiments at 24 h at 37 °C (Table 1). The complexes also demonstrated complete stability in rat serum at 24 h at 37 °C (Figure 3) with nonspecific protein binding of <10% (Table 1). No oxidation to the permetallate or colloidal (MO_2_) chemical forms was observed by radio-HPLC (method 1) or radio-TLC, respectively, nor was decomposition of the metal complex observed. The stability of these complexes is attractive for radiopharmaceutical development and provides further evidence that the [M(CO)_3_]^+^ cores form highly stable complexes with TACN-based chelators.

All successfully synthesized radiocomplexes demonstrated hydrophilic character. The **M-5** (diacid) complexes, which carry an overall −1 charge at physiological pH, demonstrated the greatest hydrophilic character, with logD_7.4_ values of around −2 (Table 1). Complexes **M-2** (diamide), carrying an overall +1 charge at physiological pH, demonstrated the next highest hydrophilicity, with logD_7.4_ values of around −1. Finally, complexes **M-3** (dialcohol), also carrying a +1 charge, demonstrated the least hydrophilic character, with logD_7.4_ values of around −0.3. For radiopharmaceutical development, hydrophilic complexes are typically preferred for their rapid clearance via the renal-urinary pathway. This results in shorter residence times of the radiopharmaceutical in the clearance organs leading to less exposure of healthy tissues to radiation and higher tumor to background ratios for activity accumulation, for improved nuclear imaging. Given their excellent stability and hydrophilic character, either chelator **2** or **3** could be conjugated to a biological targeting vector, labeled with the [M(CO)_3_]^+^ cores, and evaluated as theranostic radiopharmaceuticals. However, improvement of the RCYs would be necessary, which may be aided in future efforts via incorporation of density functional theory (DFT) computational studies.

## 4 Conclusions

TACN-based chelators bearing amide, alcohol, and ketone functional groups on their pendent arms were successfully synthesized and fully characterized. These chelators were reacted with [M(CO)_3_]^+^ cores (M = ^nat^Re, ^186^Re, ^99m^Tc). Only chelator **4** (ketone derivative) was not successfully labeled with the [M(CO)_3_]^+^ cores, in alignment with our previous investigations of TACN-based chelators.^16^ Chelator **2** (amide derivative) was labeled with the [^nat^Re(CO)_3_]^+^ and [^99m^Tc][Tc(CO)_3_]^+^ cores in moderate yields, with low yield observed for radiolabeling with the [^186^Re][Re(CO)_3_]^+^ core. Chelator **3** (alcohol derivative) was labeled with the [^nat^Re(CO)_3_]^+^ core in excellent yields and radiolabeled with the [^99m^Tc][Tc(CO)_3_]^+^ and [^186^Re][Re(CO)_3_]^+^ cores in moderate yields. The **M-2** and **M-3** complexes demonstrated hydrophilic character and were highly stable in PBS, l-cysteine, l-histidine, and rat serum through 24 h. The radiolabeling of these chelators presented some challenges, which may limit their potential for radiopharmaceutical development. Nevertheless, valuable insights were gained about the impact of the TACN pendent arms on chelator radiolabeling with the [M(CO)_3_]^+^ cores, which will inform optimization of the TACN chelator design. Future studies will include the evaluation of TACN-based chelators bearing polar and ionizable functional groups on their pendent arms, such as sulfonate and phosphonic acid pendent arms.

## Supporting Information Available

Synthesis protocols; ^1^H NMR and ^13^C NMR spectra for the chelators; ^1^H NMR, ^13^C NMR, and IR spectra for the ^nat^Re-complexes; TLC for the radiocomplexes; and crystal data (PDF)
