# Supplementary material for: [186Re]Re- and [99mTc]Tc-Tricarbonyl Metal Complexes with 1,4,7-Triazacyclononane-Based Chelators Bearing Amide, Alcohol, or Ketone Pendent Groups
Source: ACS Omega. 2024 Sep 11;9(38):39925–35. doi: 10.1021/acsomega.4c05699 (PMC11425660; doi:10.1021/acsomega.4c05699)
Supplement: Supplementary file 1 — ao4c05699_si_001.pdf [file ao4c05699_si_001.pdf]

Supporting Information

[^{186}Re]Re- and [^{99m}Tc]Tc-tricarbonyl metal complexes with 1,4,7-triazacyclononane-based chelators bearing amide, alcohol, or ketone pendent groups

Rebecca Hoerres[a§#], Ritin Kamboj[a§$], Nora Pryor[a], Steven P. Kelley[a], and Heather M. Hennkens[a,b*]

[a]Department of Chemistry, University of Missouri, 601 South College Avenue, Columbia, MO, 65211, United States

[b]Research Reactor Center, University of Missouri, 1513 Research Park Drive, Columbia, MO, 65211, United States

*Email: HennkensH@missouri.edu

[§]R.H. and R.K. contributed equally to this paper

Table of Contents

1. Synthetic Procedures. S3

2. NMR Spectra. S5

3. IR Spectra. S13

4. TLC Data. S14

5. Crystal Structure Data. S18

6. References. S29

1. Synthetic Procedures

1.1 *N*-benzyl-2-bromoacetamide

N-benzyl-2-bromoacetamide was synthesized following a literature procedure and is shown in Scheme S1.[1] Benzylamine (2.5 g, 0.023 mol) was diluted with 10 mL of dichloromethane, and the solution was cooled in an ice bath. Bromoacetyl bromide (2.3 g, 0.011 mol) in 5 mL of dichloromethane was added dropwise over 5 min. A white precipitate was immediately observed. The reaction mixture was removed from the ice bath and warmed to room temperature. After 3 h of stirring at room temperature, the reaction mixture was filtered, and the filtrate was dried under reduced pressure to yield *N*-benzyl-2-bromoacetamide as a white powder. No further purification was needed. Isolated yield: 2.0 g (76%). The product was characterized by HR-ESI-MS, ^{1}H NMR and ^{13}C NMR. Characterization data were reported previously.[2]

1.2 Alternative synthesis for *N*-benzyl-2-(1,4,7-triazonan-1-yl)acetamide (1)

Compound **1** was synthesized as previously reported and is shown in Scheme S1.[1, 2] Briefly, TACN (167 mg, 1.3 mmol) was reacted with *N,N*-dimethyl formamide dimethyl acetal (154 mg, 1.3 mmol) in 3 mL of anhydrous acetonitrile at 80 °C for 3 h. *N*-benzyl-2-bromoacetamide (300 mg, 1.3 mmol) in 5 mL of anhydrous tetrahydrofuran was added, and the reaction was stirred for 18 h at room temperature. The solvent was removed under reduced pressure, and the intermediate product was reconstituted in 10 mL of water with sodium hydroxide (120 mg, 3.0 mmol). The resulting solution was stirred at 90 °C. The reaction progress was monitored every 6-12 h by HPLC (Method 1, t_R = 6.7 min). Sodium hydroxide (30 mg, 0.75 mmol) was added, as needed, to achieve reaction completion. The final product, **1**, was purified by semi-preparative HPLC. Overall isolated yield: 180 mg (50%). The product was characterized by HR-ESI-MS, ^{1}H NMR, and ^{13}C NMR. Characterization data were reported previously.[2]

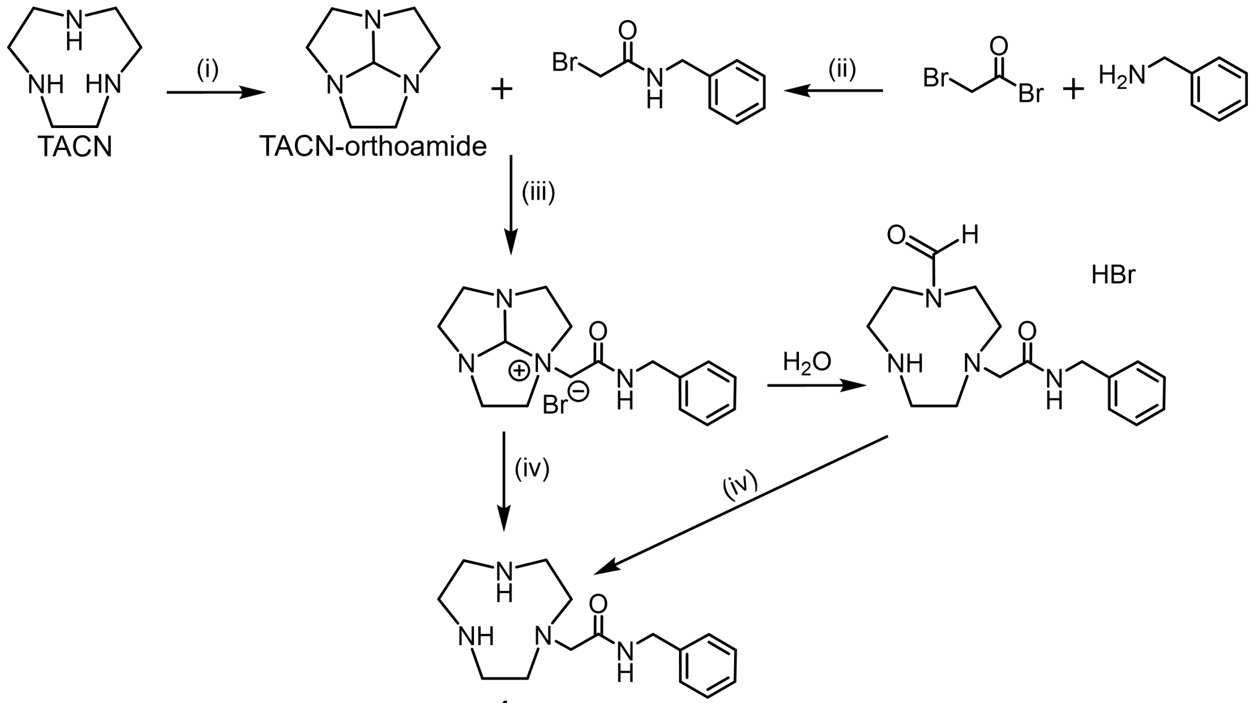

1

Scheme S1. Synthesis of *N*-benzyl-2-bromoacetamide and compound **1**. (i) *N,N*-dimethyl formamide dimethyl acetal (1 eq), acetonitrile, 80 °C, 3 h, 93% (ii) dichloromethane, 0 °C to RT, 3 h, 76%. (iii) tetrahydrofuran, RT, 18 h, 61%. (iv) sodium hydroxide (3.1 eq), water, 90 °C, 18 h, 85%.

1.3 Synthesis of the [$^{nat}Re(CO)_3(OH_2)_3$](NO_3) precursor

(NEt_4)$_2$[$Re(CO)_3Br_3$] was synthesized following an established literature procedure.[3] To a three-neck round bottom flask under nitrogen atmosphere, tetraethylammonium bromide (570 mg, 2.9 mmol) in 20 mL of diglyme was added. The reaction was brought to 80 °C in an oil bath, after which rhenium(I) pentacarbonyl bromide (500 mg, 1.2 mmol) in 20 mL of diglyme was added. The reaction was increased to a temperature of 115 °C and, once this temperature was reached, allowed to react for 4 h under nitrogen. The reaction was allowed to cool overnight. The white precipitate was filtered and washed with cold diglyme (20 mL). The resulting white precipitate was recovered and slurried with anhydrous ethanol (20 mL) to remove any excess tetraethylammonium bromide. The slurry was filtered, and the recovered product was washed with diglyme (10 mL) and cold diethyl ether (20 mL). The product, (NEt_4)$_2$[$Re(CO)_3Br_3$], was dried under vacuum overnight. Isolated yield: 801 mg (84%). IR spectroscopy was used to confirm the anticipated structure via detection of the CO ligand stretching bands at 1847 cm^{-1} and 1996 cm^{-1}, which matched the values reported in literature.[3] The (NEt_4)$_2$[$Re(CO)_3Br_3$] was stored in a desiccator and converted, as needed, to [$Re(CO)_3(OH_2)_3$](NO_3) by reacting (NEt_4)$_2$[$Re(CO)_3Br_3$] with silver nitrate (3 molar equivalents) in water at RT for 30 min followed by filtering to remove

the precipitated silver bromide. The filtrate contained the product, $[Re(CO)_3(OH_2)_3](NO_3)$, which was used without further purification.

2. NMR Spectra

Figure S1. ^{1}H NMR of **1** (300 MHz, D_2O), synthesized by reaction of TACN with *N*-benzyl-2-bromoacetamide

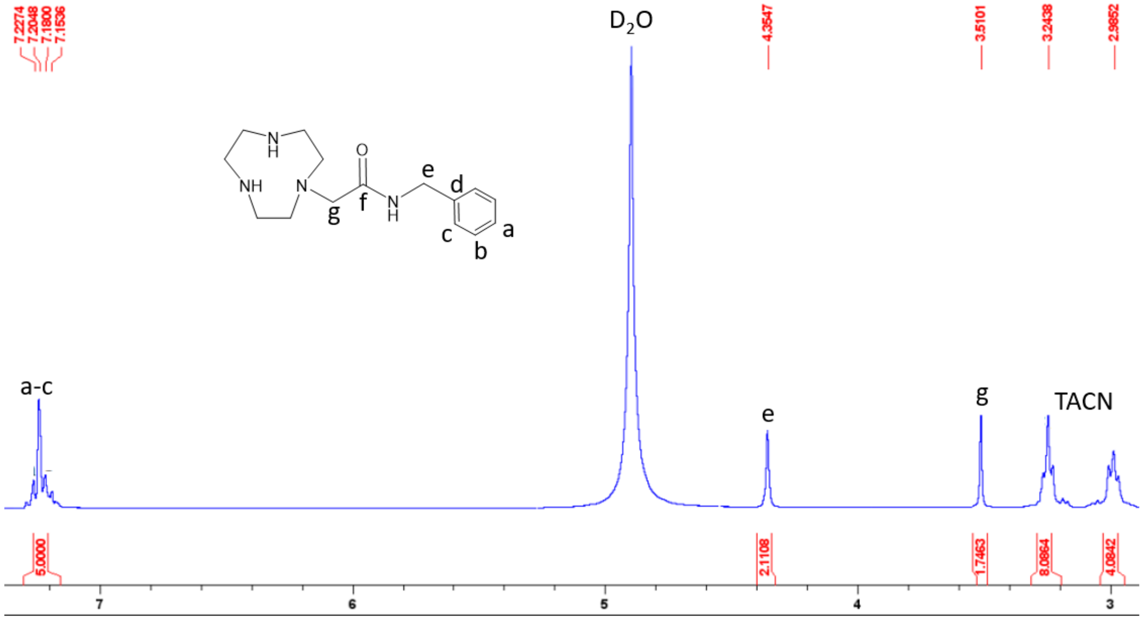

Figure S2. ^{1}H NMR of **2** (600 MHz, CD_3CN)

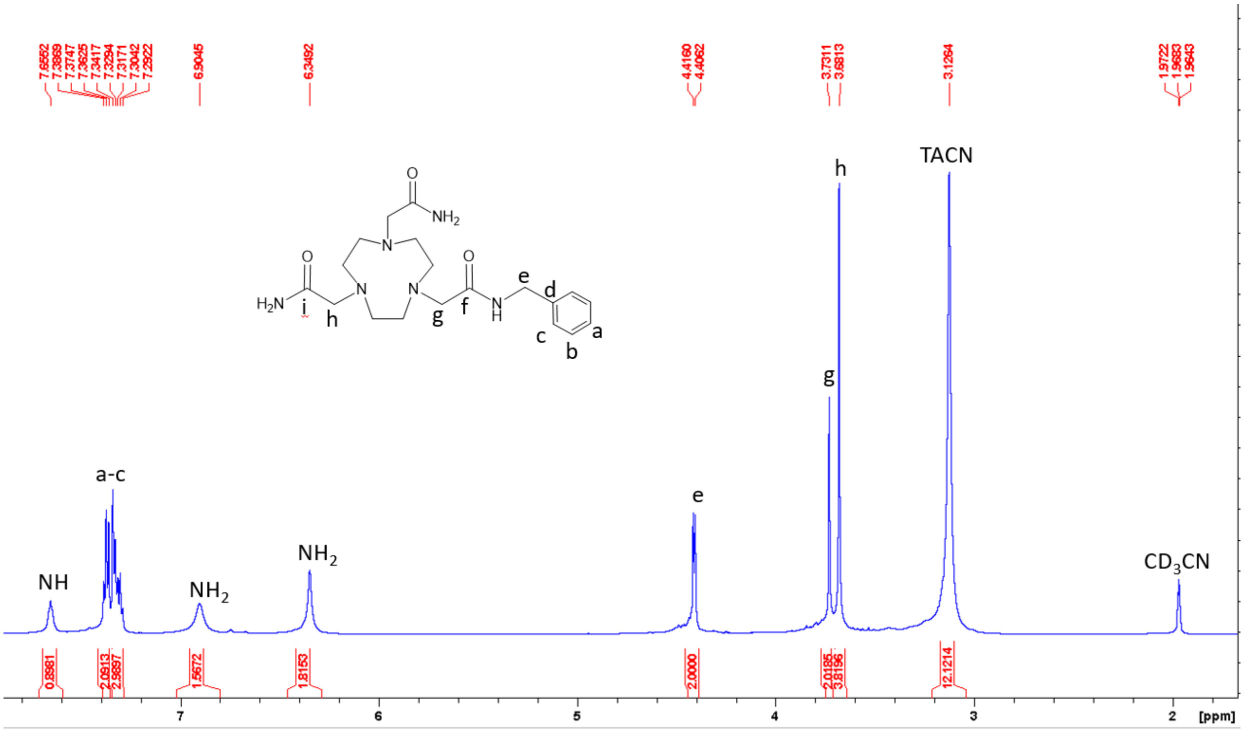

Figure S3. ^{13}C NMR of **2** (600 MHz, CD_3CN)

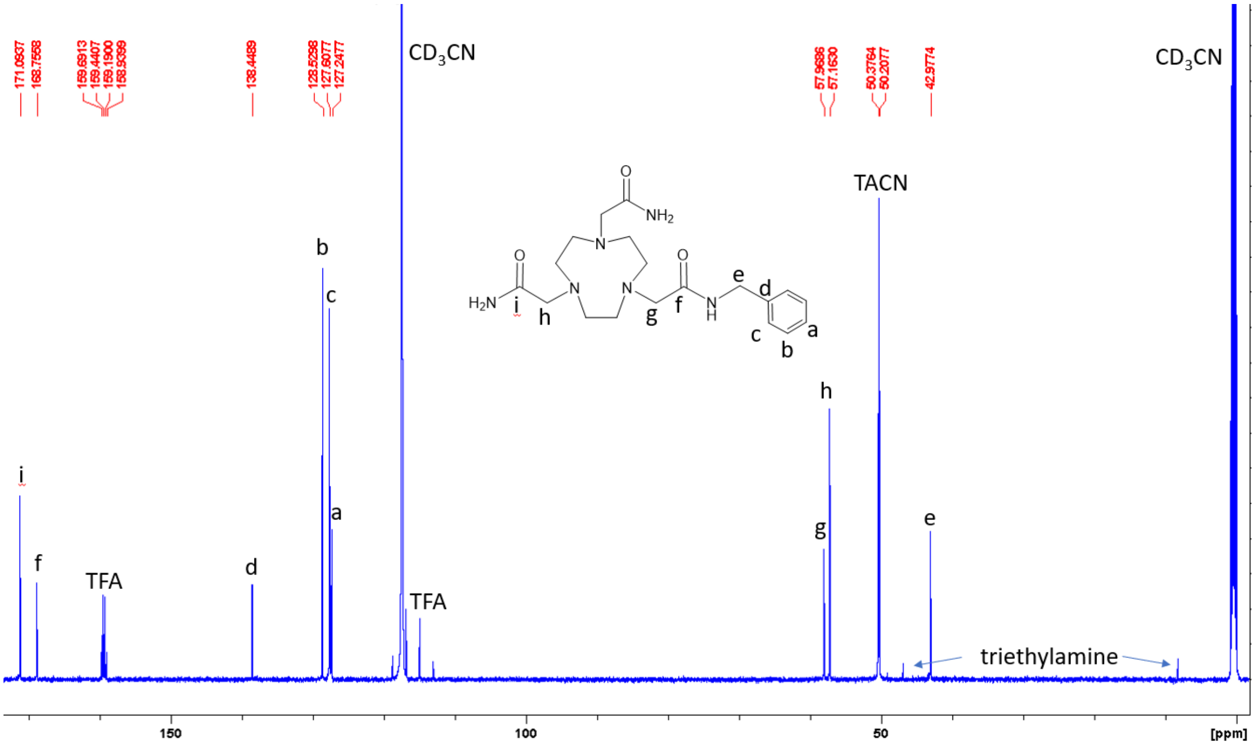

Figure S4. ^{1}H NMR of **3** (600 MHz, D_2O)

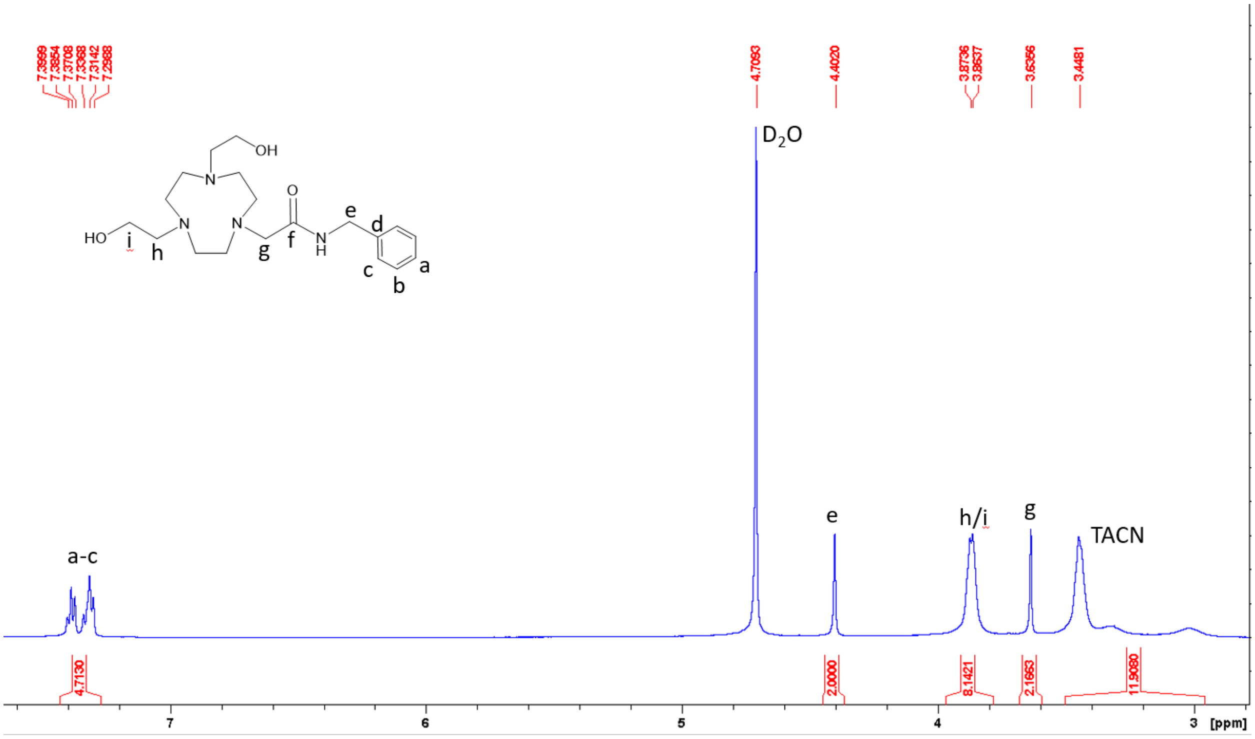

Figure S5. ^{13}C NMR of **3** (600 MHz, CD_3CN)

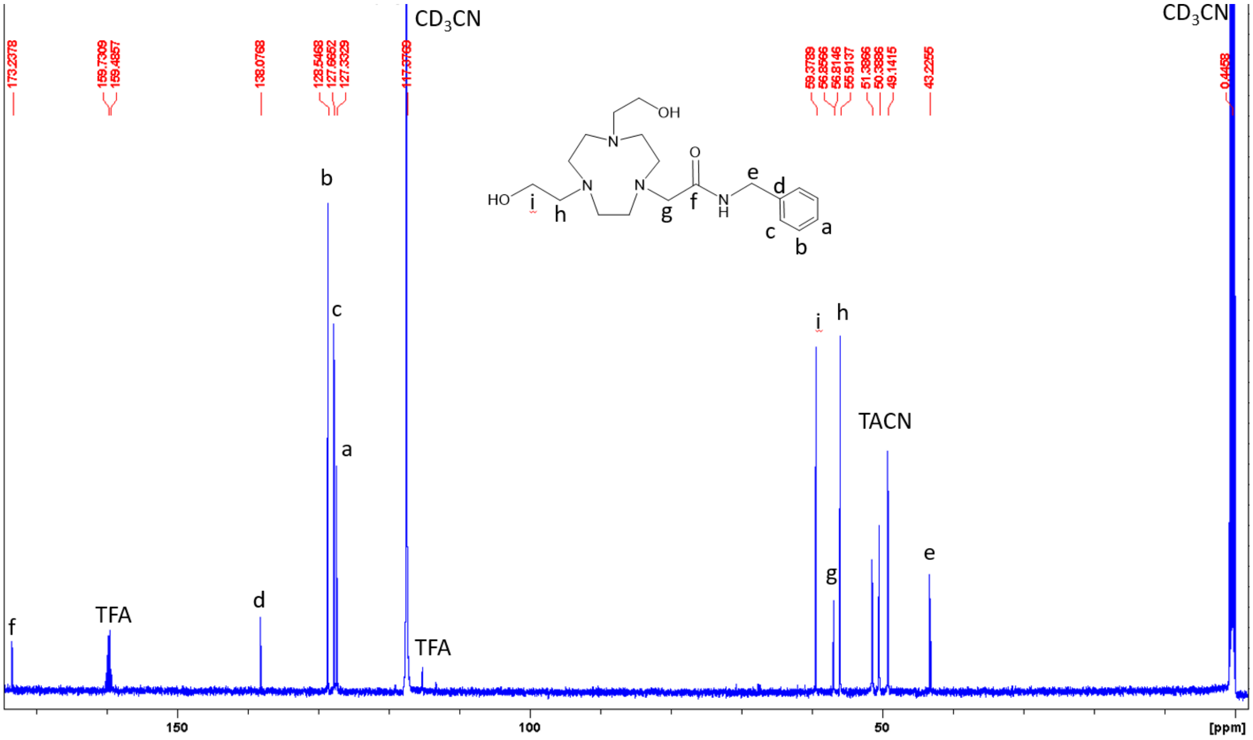

Figure S6. ^{1}H NMR of **4** (600 MHz, $(CD_3)_2SO$)

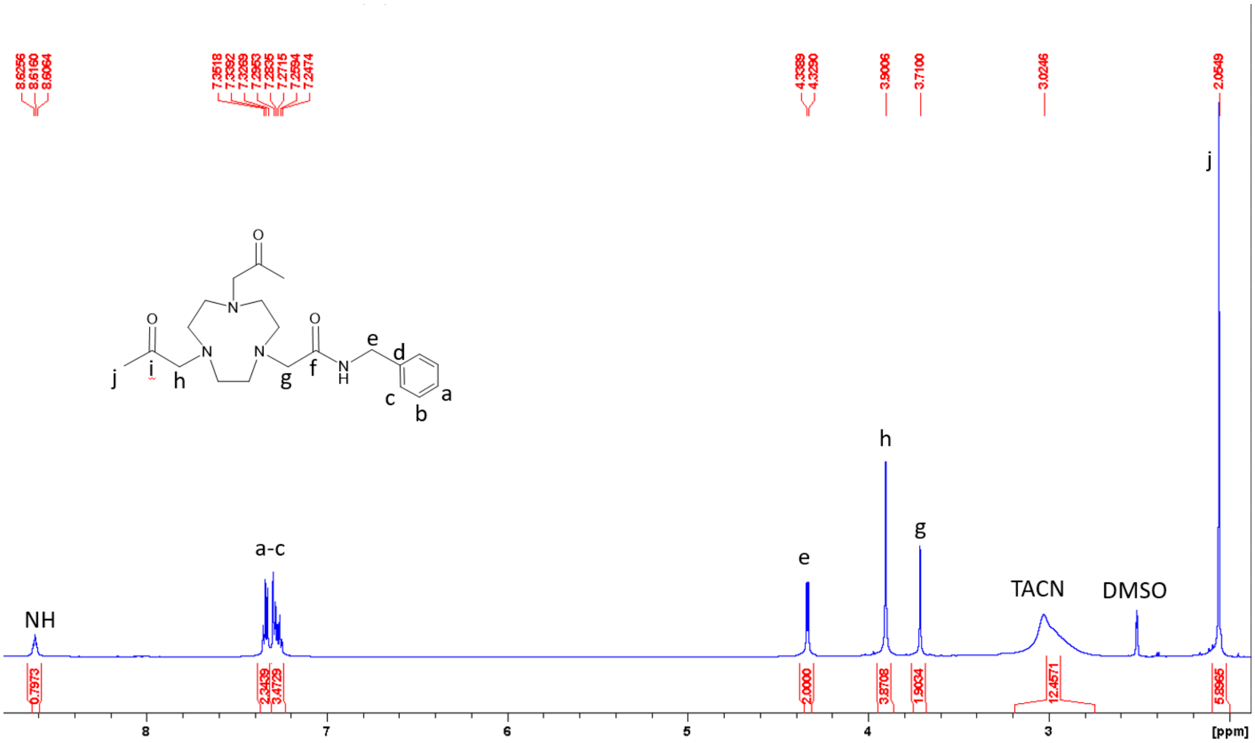

Figure S7. ^{13}C NMR of **4** (600 MHz, $(CD_3)_2SO$)

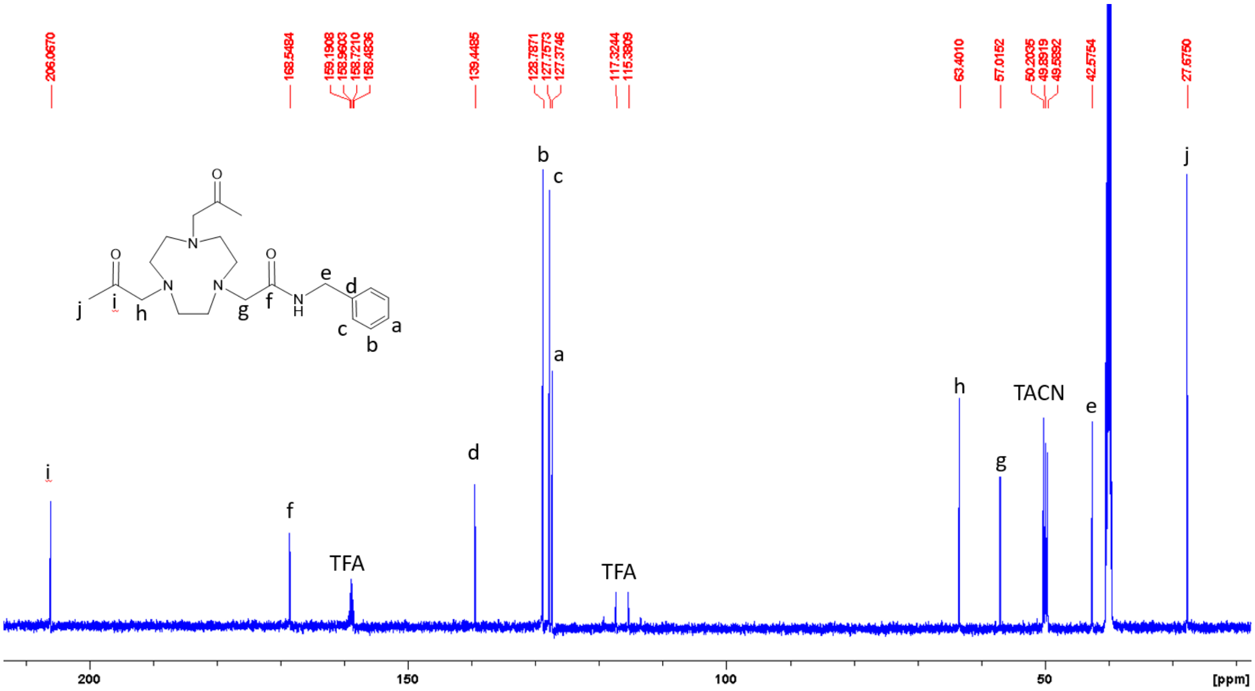

Figure S8. ^{1}H NMR of **5** (600 MHz, $(CD_3)_2SO$)

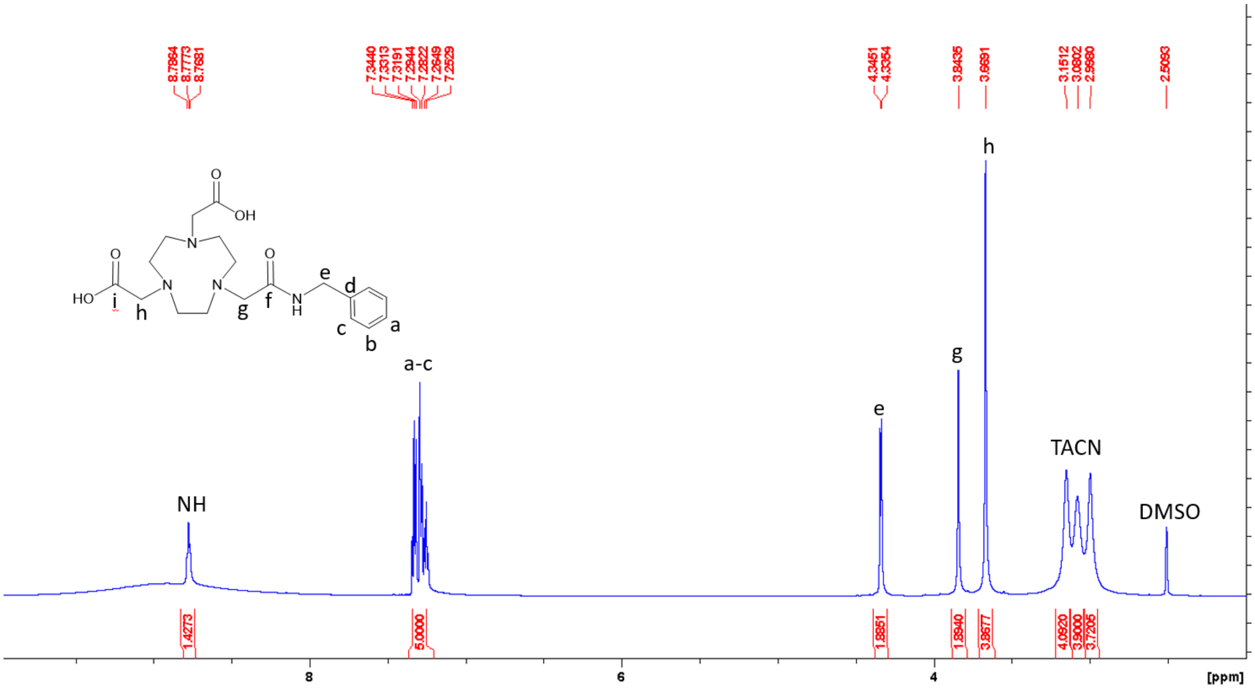

Figure S9. ^{13}C NMR of **5** (600 MHz, $(CD_3)_2SO$)

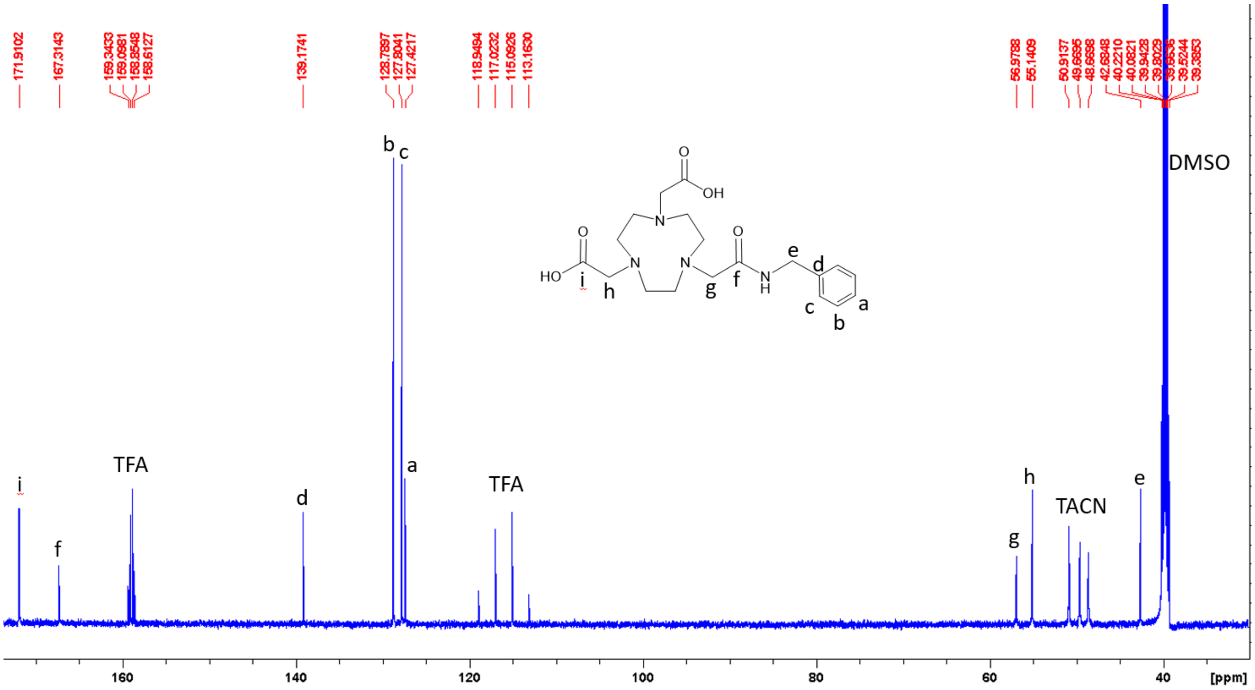

Figure S10. ^{1}H NMR of **Re-2** (600 MHz, CD_3CN)

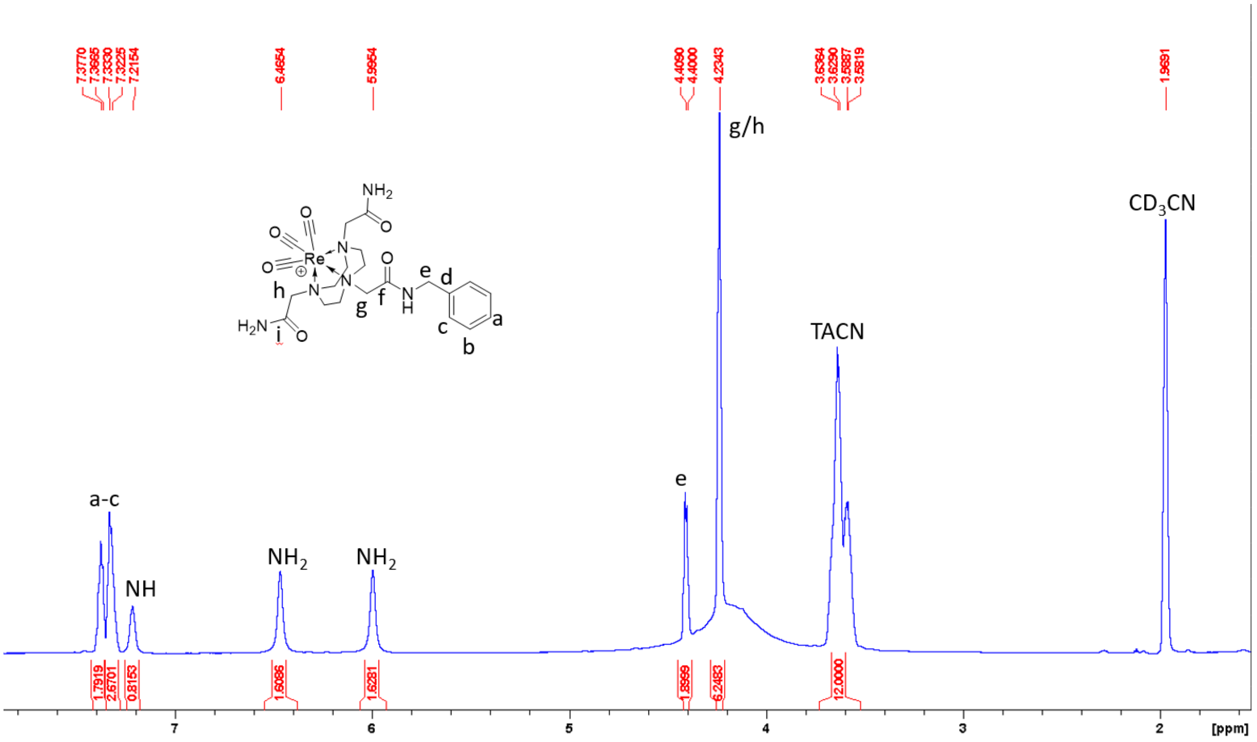

Figure S11. ^{13}C NMR of **Re-2** (600 MHz, CD_3CN)

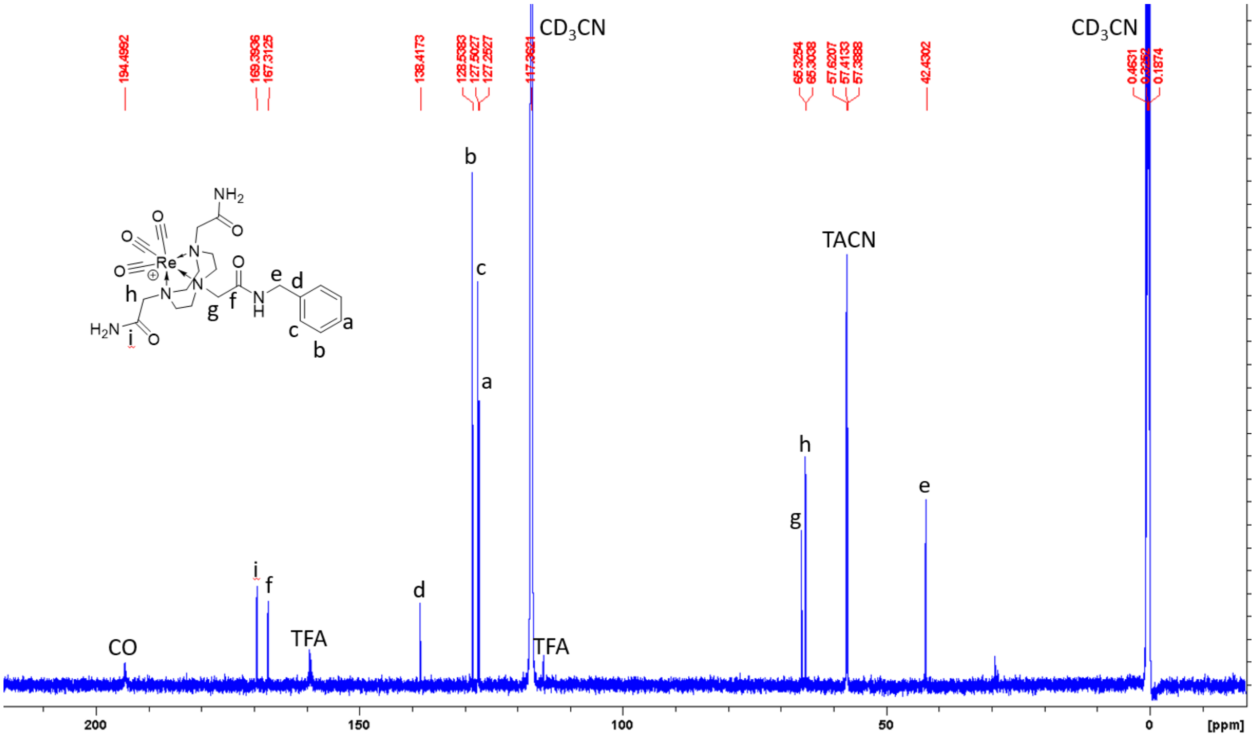

Figure S12. ^{1}H NMR of **Re-3** (600 MHz, CD_3CN)

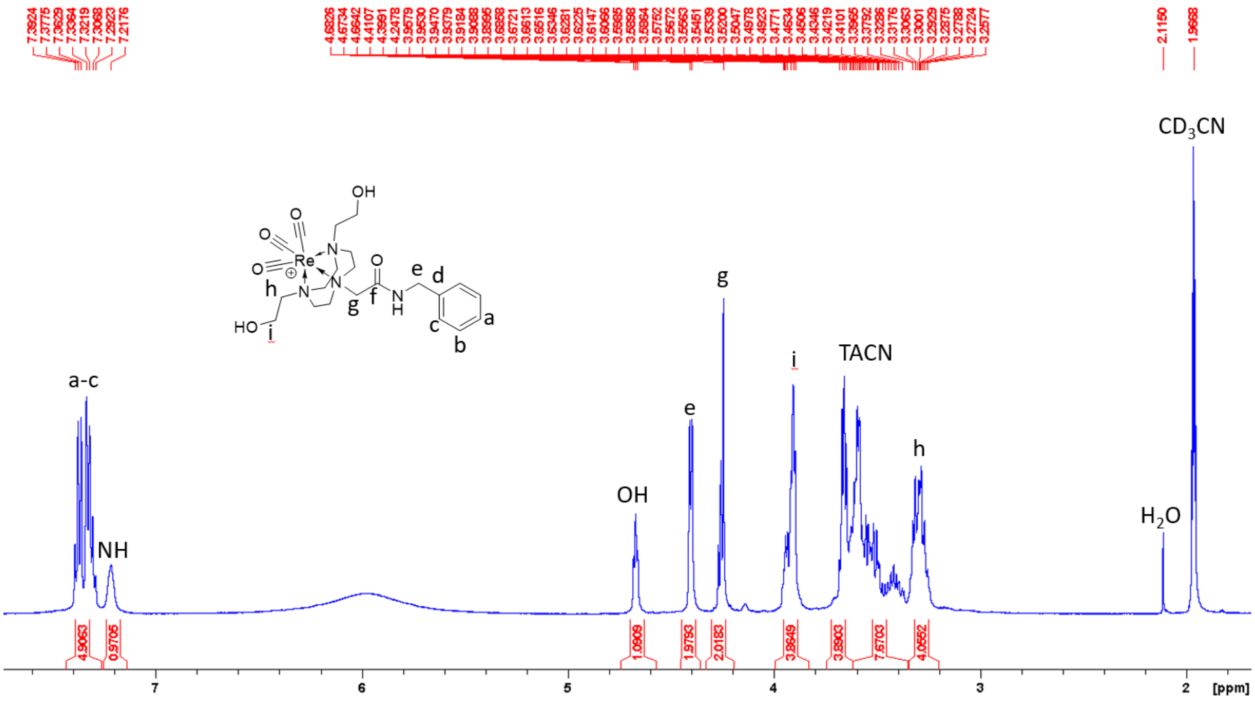

Figure S13. ^{13}C NMR of **Re-3** (600 MHz, CD_3CN)

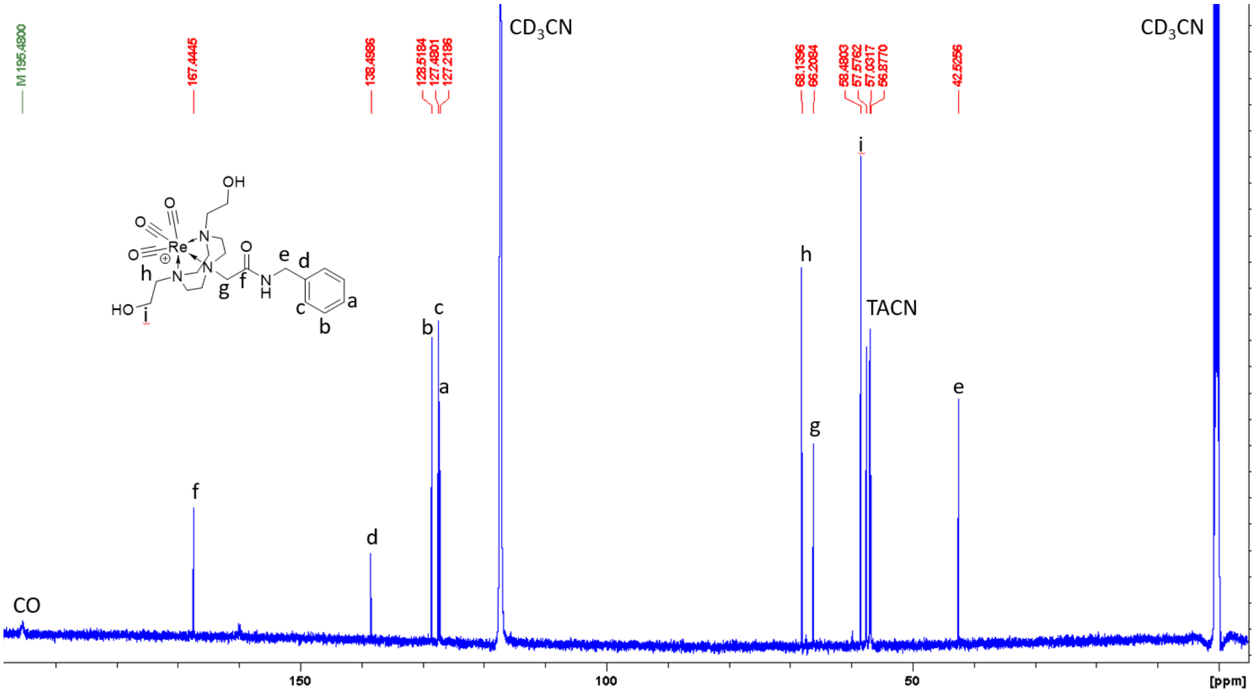

Figure S14. ^{1}H NMR of **Re-5** (600 MHz, CD_3CN)

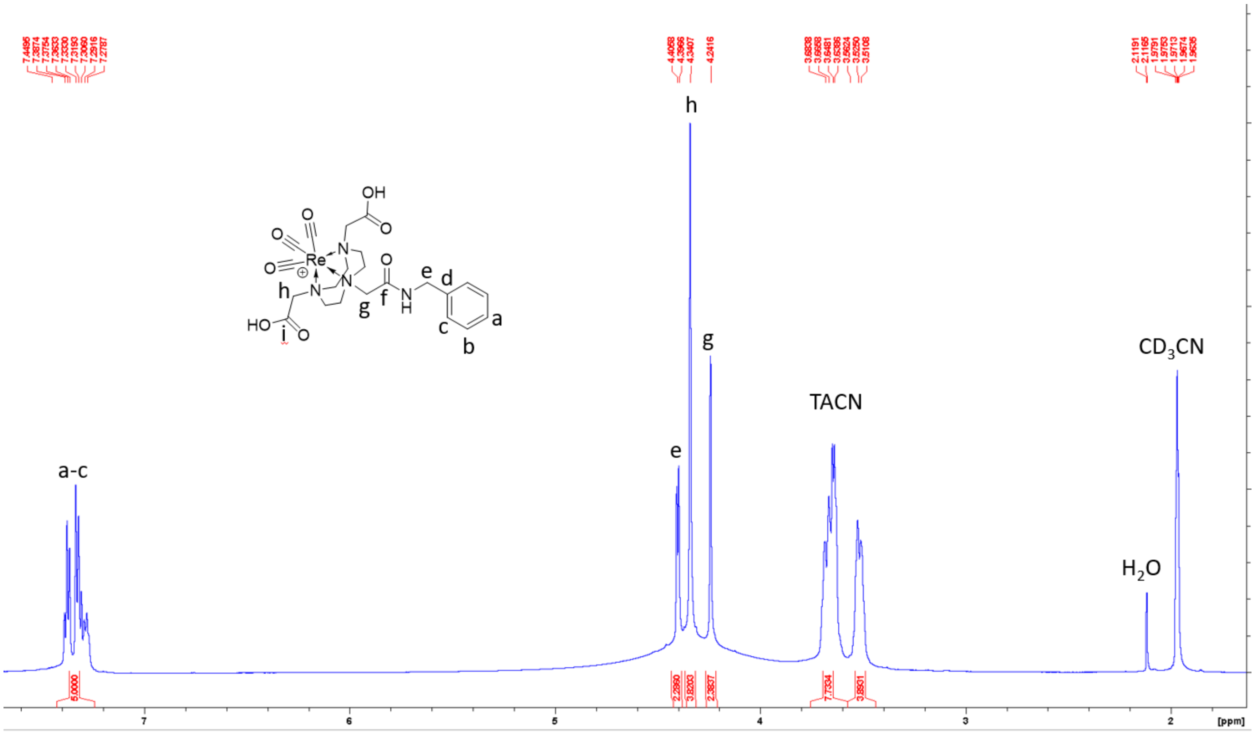

Figure S15. ^{13}C NMR of **Re-5** (600 MHz, CD_3CN)

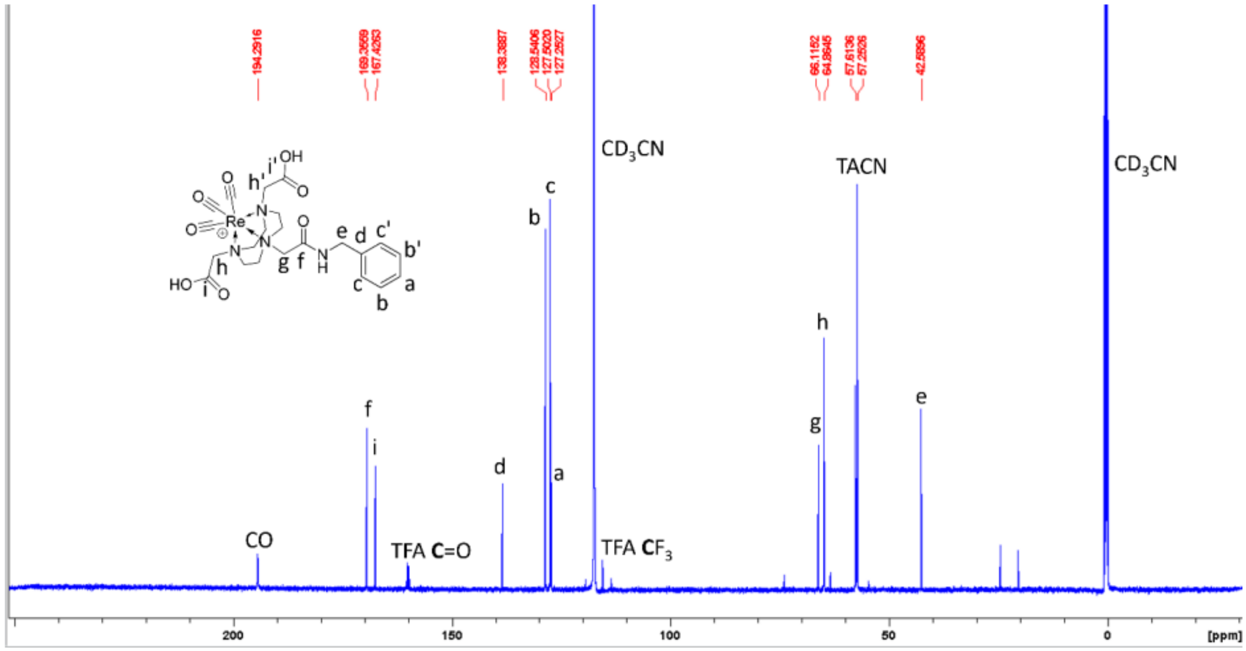

3. IR Spectra

Figure S16. Re-2

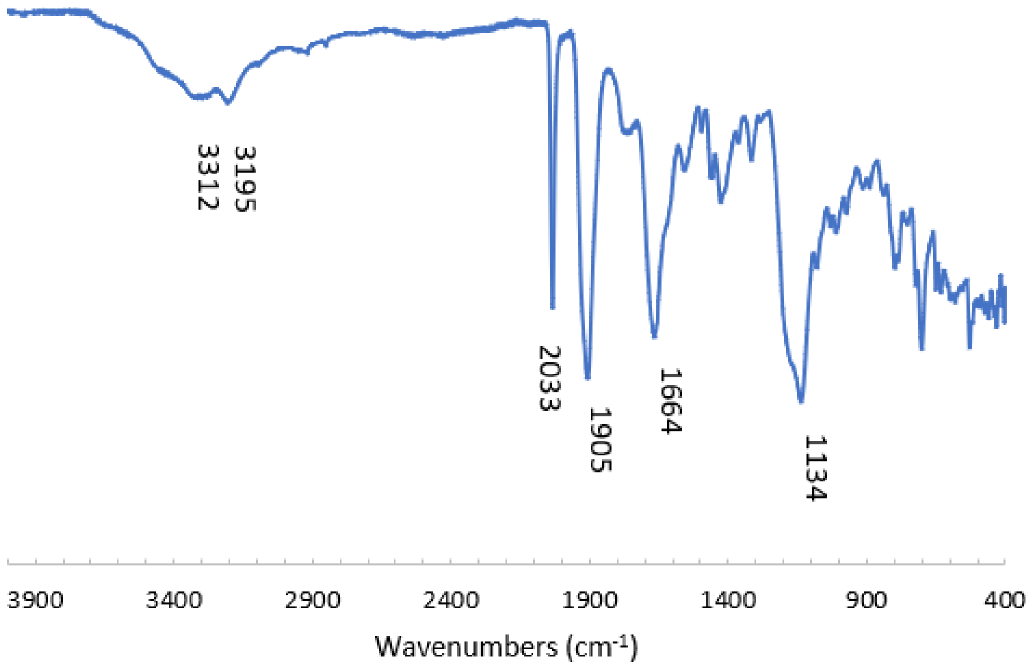

Figure S17. Re-3

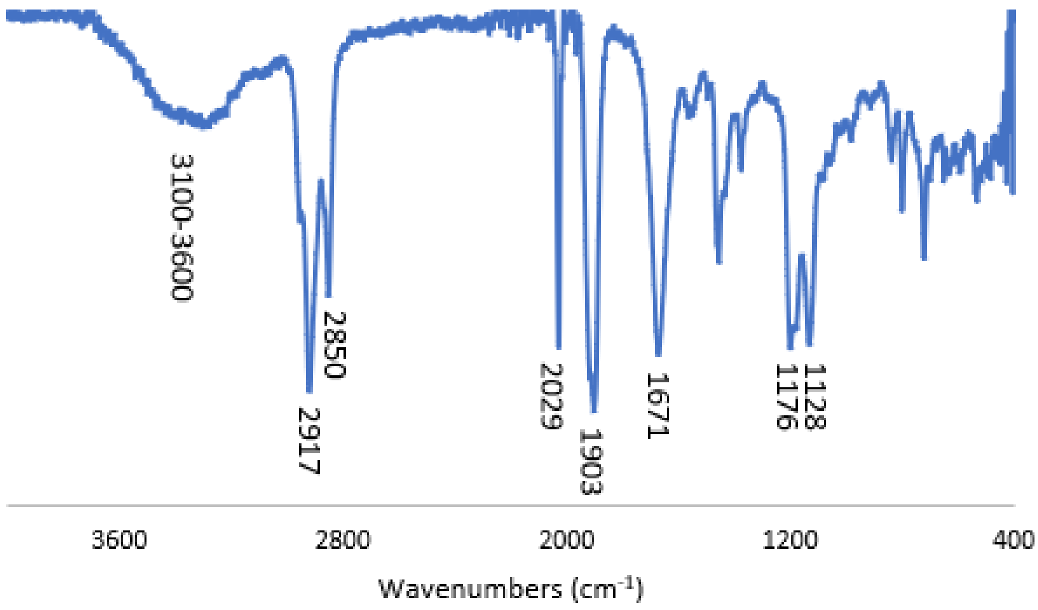

Figure S18. Re-5

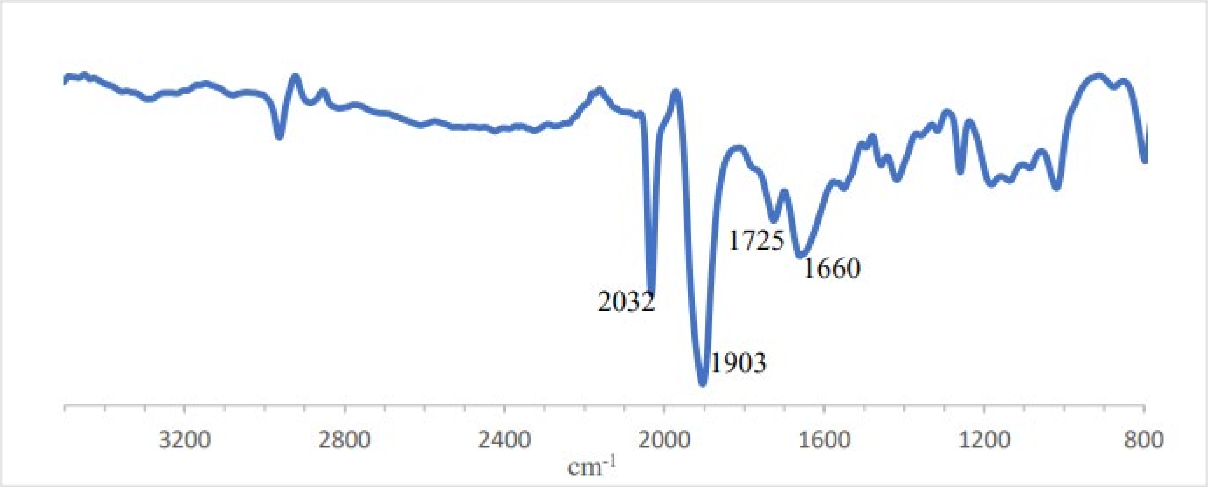

4. TLC Data

Figure S19. Representative radioTLC for [^{99m}Tc]Tc-2 stability evaluation

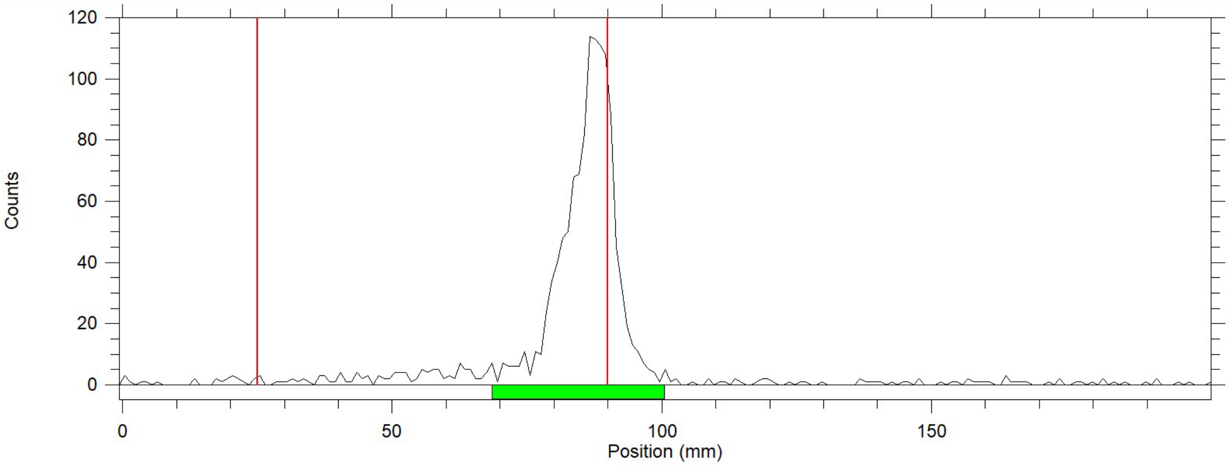

Figure S20. Representative radioTLC for [^{99m}Tc]Tc-3 stability evaluation

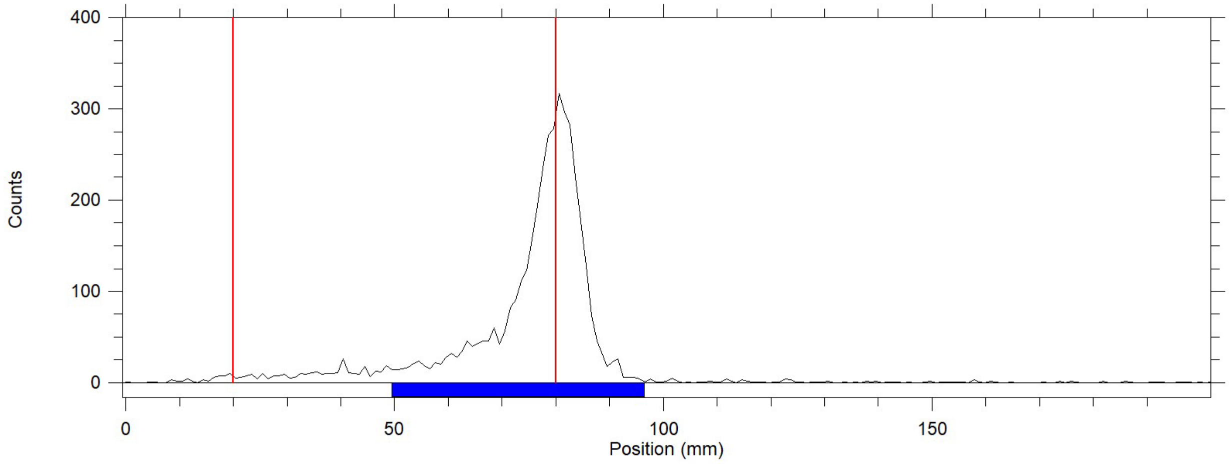

Figure S21. Representative radioTLC for [^{99m}Tc]Tc-5 stability evaluation (origin: 50 mm, solvent front: 125 mm)

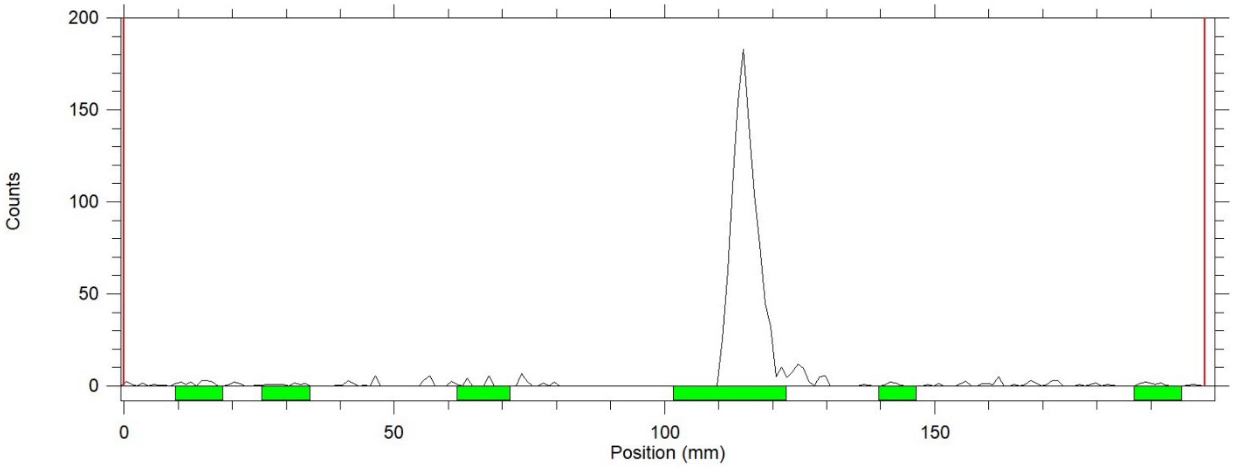

Figure S22. Representative radioTLC for [^{186}Re]Re-2 stability evaluation

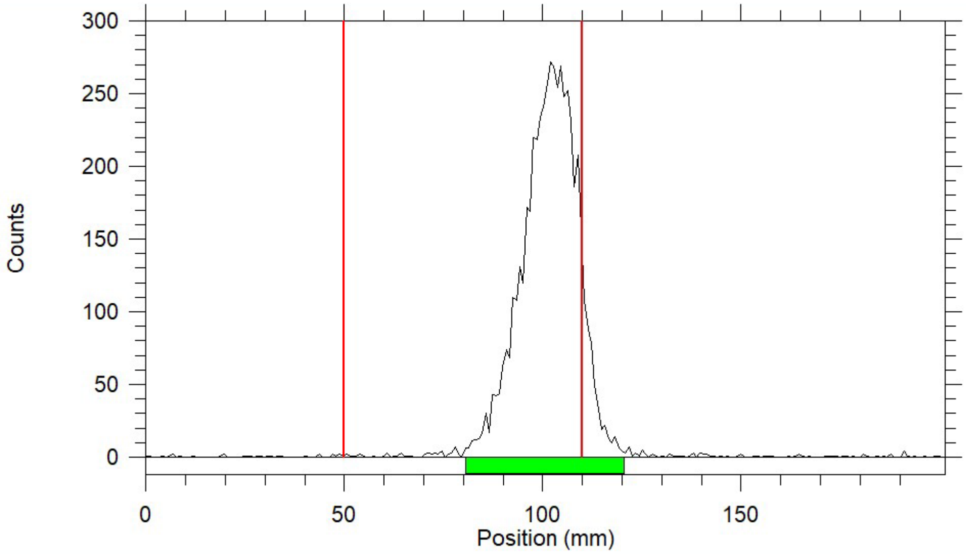

Figure S23. Representative radioTLC for [^{186}Re]Re-3 stability evaluation

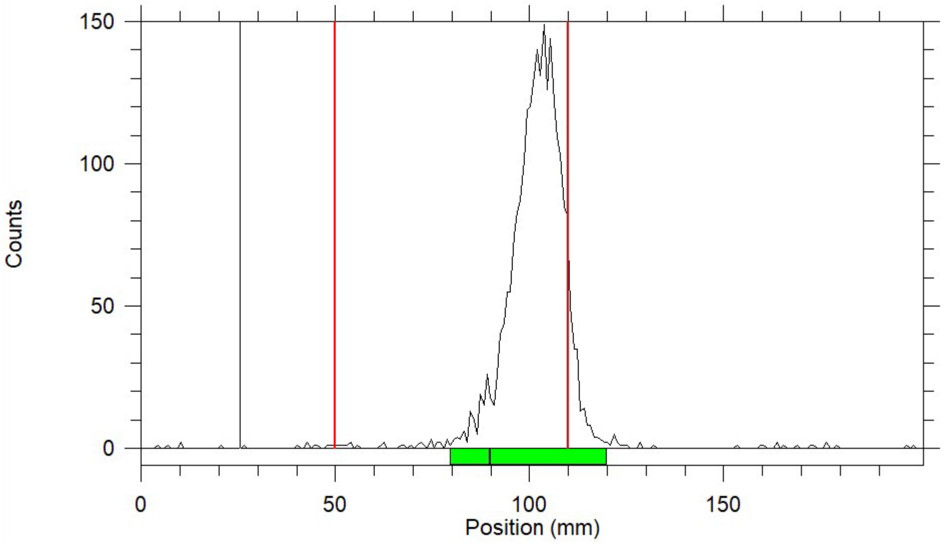

Figure S24. Representative radioTLC for [^{186}Re]Re-5 stability evaluation

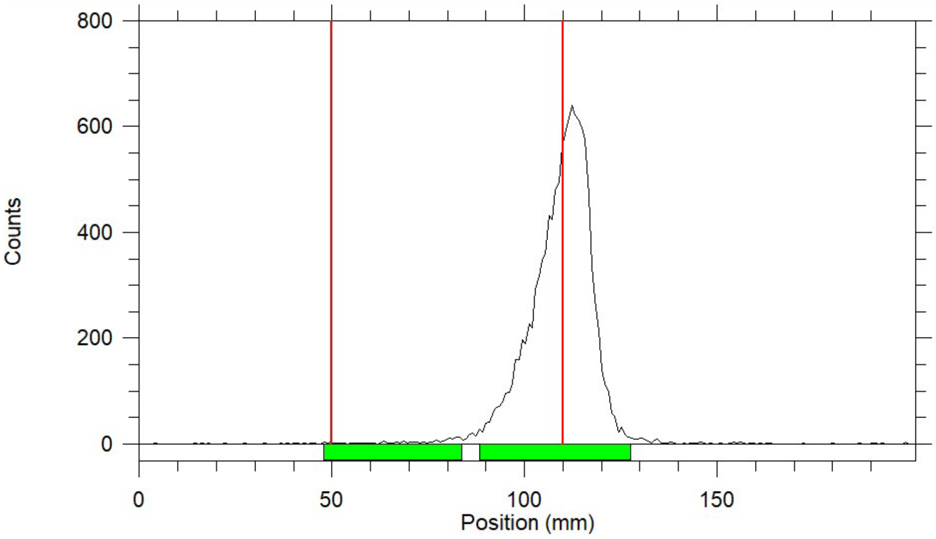

5. Crystal Structure Data

Table S1. Crystal data and structure refinement.

Empirical formula	$C_{28}H_{36}N_4O_{10}F_3Re$
Formula weight	831.81
Temperature	173.0 K
Wavelength	0.71073 Å
Crystal system	Monoclinic
Space group	$P2_1/n$
Unit cell dimensions	a = 12.7621(7) Å, α = 90°
	b = 17.1038(9) Å, β = 110.0536(18)°
	c = 15.5215(8) Å, γ = 90°
Volume	3182.6(3) $Å^3$
Z	4
Density (calculated)	1.736 Mg/m^3
Absorption coefficient	3.896 mm^{-1}
F(000)	1656
Crystal size	0.25 x 0.16 x 0.14 mm^3
Theta range for data collection	2.543 to 29.590°
Index ranges	-17<=h<=17, -23<=k<=23, -21<=l<=21
Reflections collected	82844
Independent reflections	8931 [R(int) = 0.0378]
Completeness to theta = 25.242°	99.9 %
Absorption correction	Semi-empirical from equivalents
Max. and min. transmission	0.7459 and 0.5786
Refinement method	Full-matrix least-squares on F^2
Data / restraints / parameters	8931 / 1 / 447
Goodness-of-fit on F^2	1.082
Final R indices [I>2sigma(I)]	R1 = 0.0339, wR2 = 0.0671
R indices (all data)	R1 = 0.0459, wR2 = 0.0765
Largest diff. peak and hole	2.810 and -1.400 e.$Å^{-3}$

Table S2. Atomic coordinates (×10^4) and equivalent isotropic displacement parameters (Å^2×10^3). U(eq) is defined as one third of the trace of the orthogonalized Uij tensor.

	x	y	z	U (eq)
Re(1)	976(1)	194(1)	2823(1)	25(1)
F(1S)	-611(5)	2047(3)	833(3)	108(2)
F(2S)	-157(5)	3231(3)	597(5)	159(3)
F(3S)	-1413(4)	2573(4)	-438(4)	125(2)
O(1)	3842(3)	246(2)	1202(2)	50(1)
O(1S)	397(4)	1543(2)	-362(3)	82(2)
O(2)	1018(3)	-2798(2)	2658(3)	47(1)
O(2S)	1167(5)	2701(3)	-177(4)	94(2)
O(3)	-810(2)	-2684(2)	2418(2)	40(1)
O(4)	4117(3)	153(2)	5946(2)	45(1)
O(5)	3040(3)	1034(2)	6304(2)	50(1)
O(6)	-1002(3)	272(2)	1014(2)	56(1)
O(7)	-690(3)	151(2)	3846(3)	52(1)
O(8)	1002(3)	1989(2)	2898(2)	51(1)
N(1)	2320(3)	120(2)	2214(2)	28(1)
N(2)	1225(3)	-1106(2)	2838(2)	27(1)
N(3)	2469(2)	73(2)	4090(2)	25(1)
N(4)	2650(4)	1028(3)	162(3)	50(1)
C(1)	2053(6)	-648(3)	1695(4)	31(1)
C(1')	2658(12)	-737(8)	2109(10)	30(3)
C(1S)	506(5)	2242(4)	-128(4)	61(2)
C(2)	1898(4)	-1291(3)	2254(4)	47(1)
C(2S)	-403(6)	2499(4)	209(4)	70(2)
C(3)	1820(5)	-1297(3)	3825(3)	59(2)
C(4)	2645(5)	-768(3)	4336(3)	63(2)
C(5)	3372(6)	500(4)	3800(5)	21(1)

	x	y	z	U (eq)
C(5')	3527(10)	118(9)	3941(8)	38(3)
C(6)	3415(8)	135(5)	2904(7)	23(2)
C(6')	3397(14)	420(9)	3043(11)	33(3)
C(7)	2174(4)	713(3)	1479(3)	40(1)
C(8)	2988(4)	627(3)	940(3)	39(1)
C(9)	3246(5)	1001(4)	-486(3)	57(2)
C(10)	4307(5)	1485(3)	-200(3)	47(1)
C(11)	5297(5)	1151(3)	-168(4)	54(1)
C(12)	6261(5)	1591(4)	57(4)	61(2)
C(13)	6232(5)	2377(4)	250(4)	59(2)
C(14)	5243(6)	2722(4)	201(4)	65(2)
C(15)	4276(5)	2273(4)	-20(4)	60(2)
C(16)	2329(3)	540(2)	4840(2)	30(1)
C(17)	3287(3)	536(2)	5752(2)	29(1)
C(18)	3886(5)	1181(4)	7208(3)	62(2)
C(19)	3627(5)	1962(3)	7510(3)	59(1)
C(20)	140(3)	-1516(2)	2525(3)	37(1)
C(21)	203(3)	-2410(2)	2553(3)	33(1)
C(22)	-915(4)	-3538(3)	2348(4)	47(1)
C(23)	-2103(4)	-3732(3)	2214(5)	66(2)
C(24)	-257(3)	236(2)	1681(3)	36(1)
C(25)	-81(3)	173(3)	3448(3)	32(1)
C(26)	971(3)	1325(2)	2859(3)	30(1)

Table S3. Bond lengths.

Bond lengths (Å)			
Re(1)-N(1)	2.228(3)	N(2)-C(3)	1.494(5)
Re(1)-N(2)	2.246(3)	N(2)-C(20)	1.477(5)
Re(1)-N(3)	2.230(3)	N(3)-C(4)	1.484(6)
Re(1)-C(24)	1.927(4)	N(3)-C(5)	1.556(7)
Re(1)-C(25)	1.912(4)	N(3)-C(5')	1.448(12)
Re(1)-C(26)	1.935(4)	N(3)-C(16)	1.473(5)
F(1S)-C(2S)	1.335(8)	N(4)-C(8)	1.326(6)
F(2S)-C(2S)	1.377(8)	N(4)-C(9)	1.455(6)
F(3S)-C(2S)	1.341(8)	C(1)-C(2)	1.455(7)
O(1)-C(8)	1.214(6)	C(1')-C(2)	1.427(14)
O(1S)-C(1S)	1.243(7)	C(1S)-C(2S)	1.494(9)
O(2)-C(21)	1.197(5)	C(3)-C(4)	1.408(7)
O(2S)-C(1S)	1.173(7)	C(5)-C(6)	1.543(11)
O(3)-C(21)	1.323(5)	C(5')-C(6')	1.44(2)
O(3)-C(22)	1.468(5)	C(7)-C(8)	1.547(6)
O(4)-C(17)	1.193(5)	C(9)-C(10)	1.518(7)
O(5)-C(17)	1.321(5)	C(10)-C(11)	1.372(8)
O(5)-C(18)	1.470(5)	C(10)-C(15)	1.380(8)
O(6)-C(24)	1.142(5)	C(11)-C(12)	1.382(8)
O(7)-C(25)	1.148(5)	C(12)-C(13)	1.380(8)
O(8)-C(26)	1.137(5)	C(13)-C(14)	1.372(9)
N(1)-C(1)	1.518(6)	C(14)-C(15)	1.392(8)
N(1)-C(1')	1.552(13)	C(16)-C(17)	1.520(5)
N(1)-C(6)	1.440(11)	C(18)-C(19)	1.490(8)
N(1)-C(6')	1.611(15)	C(20)-C(21)	1.531(6)
N(1)-C(7)	1.489(5)	C(22)-C(23)	1.496(7)
N(2)-C(2)	1.481(5)		

Table S4. Bond angles.

Bond Angles (°)			
N(1)-Re(1)-N(2)	79.69(12)	C(2)-C(1')-N(1)	112.5(9)
N(1)-Re(1)-N(3)	79.57(11)	O(1S)-C(1S)-C(2S)	111.3(6)
N(3)-Re(1)-N(2)	79.83(11)	O(2S)-C(1S)-O(1S)	129.5(7)
C(24)-Re(1)-N(1)	96.68(16)	O(2S)-C(1S)-C(2S)	119.1(6)
C(24)-Re(1)-N(2)	96.59(15)	C(1)-C(2)-N(2)	115.4(4)
C(24)-Re(1)-N(3)	175.18(16)	C(1')-C(2)-N(2)	121.5(6)
C(24)-Re(1)-C(26)	88.82(17)	F(1S)-C(2S)-F(2S)	106.4(6)
C(25)-Re(1)-N(1)	173.44(14)	F(1S)-C(2S)-F(3S)	102.7(7)
C(25)-Re(1)-N(2)	95.66(15)	F(1S)-C(2S)-C(1S)	117.1(6)
C(25)-Re(1)-N(3)	95.11(14)	F(2S)-C(2S)-C(1S)	109.4(6)
C(25)-Re(1)-C(24)	88.42(18)	F(3S)-C(2S)-F(2S)	105.0(6)
C(25)-Re(1)-C(26)	89.66(18)	F(3S)-C(2S)-C(1S)	115.3(5)
C(26)-Re(1)-N(1)	94.55(15)	C(4)-C(3)-N(2)	117.2(4)
C(26)-Re(1)-N(2)	172.50(14)	C(3)-C(4)-N(3)	117.3(4)
C(26)-Re(1)-N(3)	94.47(14)	C(6)-C(5)-N(3)	109.3(5)
C(21)-O(3)-C(22)	115.1(3)	C(6')-C(5')-N(3)	111.6(11)
C(17)-O(5)-C(18)	118.0(3)	N(1)-C(6)-C(5)	110.6(7)
C(1)-N(1)-Re(1)	102.0(3)	C(5')-C(6')-N(1)	116.2(12)
C(1')-N(1)-Re(1)	112.3(5)	N(1)-C(7)-C(8)	114.7(4)
C(1')-N(1)-C(6')	101.1(8)	O(1)-C(8)-N(4)	123.8(4)
C(6)-N(1)-Re(1)	112.1(4)	O(1)-C(8)-C(7)	124.2(4)
C(6)-N(1)-C(1)	113.2(5)	N(4)-C(8)-C(7)	112.0(4)
C(6)-N(1)-C(7)	113.1(5)	N(4)-C(9)-C(10)	113.9(4)
C(6')-N(1)-Re(1)	102.3(6)	C(11)-C(10)-C(9)	120.1(5)
C(7)-N(1)-Re(1)	112.0(3)	C(11)-C(10)-C(15)	119.3(5)
C(7)-N(1)-C(1)	103.7(3)	C(15)-C(10)-C(9)	120.5(5)
C(7)-N(1)-C(1')	121.9(6)	C(10)-C(11)-C(12)	120.8(5)

Bond Angles (°)			
C(7)-N(1)-C(6')	104.1(6)	C(13)-C(12)-C(11)	119.9(6)
C(2)-N(2)-Re(1)	108.3(2)	C(14)-C(13)-C(12)	119.9(5)
C(2)-N(2)-C(3)	112.6(4)	C(13)-C(14)-C(15)	119.9(6)
C(3)-N(2)-Re(1)	104.3(3)	C(10)-C(15)-C(14)	120.2(6)
C(20)-N(2)-Re(1)	110.6(2)	N(3)-C(16)-C(17)	117.2(3)
C(20)-N(2)-C(2)	111.9(3)	O(4)-C(17)-O(5)	125.4(4)
C(20)-N(2)-C(3)	108.9(3)	O(4)-C(17)-C(16)	126.8(4)
C(4)-N(3)-Re(1)	109.0(2)	O(5)-C(17)-C(16)	107.8(3)
C(4)-N(3)-C(5)	117.9(4)	O(5)-C(18)-C(19)	106.5(4)
C(5)-N(3)-Re(1)	101.2(3)	N(2)-C(20)-C(21)	115.5(3)
C(5')-N(3)-Re(1)	114.6(5)	O(2)-C(21)-O(3)	125.5(4)
C(5')-N(3)-C(4)	91.4(7)	O(2)-C(21)-C(20)	126.4(4)
C(5')-N(3)-C(16)	118.1(6)	O(3)-C(21)-C(20)	108.1(4)
C(16)-N(3)-Re(1)	110.3(2)	O(3)-C(22)-C(23)	107.0(4)
C(16)-N(3)-C(4)	111.7(4)	O(6)-C(24)-Re(1)	178.4(5)
C(16)-N(3)-C(5)	106.1(4)	O(7)-C(25)-Re(1)	177.9(4)
C(8)-N(4)-C(9)	121.9(5)	O(8)-C(26)-Re(1)	177.8(4)
C(2)-C(1)-N(1)	112.9(4)		

Table S5. Anisotropic displacement parameters (Å^2×10^3).
The anisotropic displacement factor exponent takes the form: -2p^2[h^2 a*^{2}U^{11} + ... + 2 h k a* b* U^{12}].

	U^{11}	U^{22}	U^{33}	U^{23}	U^{13}	U^{12}
Re(1)	19(1)	27(1)	24(1)	2(1)	3(1)	0(1)
F(1S)	150(4)	95(3)	100(3)	27(3)	72(3)	1(3)
F(2S)	159(5)	104(4)	239(7)	-102(5)	101(5)	-16(4)
F(3S)	80(3)	177(6)	110(4)	2(4)	20(3)	55(3)
O(1)	67(2)	44(2)	55(2)	1(2)	40(2)	2(2)
O(1S)	113(4)	48(2)	54(2)	-13(2)	-12(2)	14(2)
O(2)	34(2)	30(2)	69(2)	4(2)	8(2)	-1(1)
O(2S)	93(4)	77(3)	123(5)	-26(3)	50(3)	-17(3)
O(3)	35(2)	34(2)	50(2)	-10(1)	14(1)	-12(1)
O(4)	37(2)	50(2)	35(2)	-10(1)	-5(1)	16(1)
O(5)	42(2)	72(2)	28(2)	-17(2)	1(1)	19(2)
O(6)	47(2)	48(2)	48(2)	7(2)	-19(2)	-5(2)
O(7)	34(2)	72(2)	59(2)	15(2)	24(2)	11(2)
O(8)	55(2)	32(2)	56(2)	-2(2)	9(2)	6(2)
N(1)	33(2)	28(2)	26(2)	-1(1)	13(1)	-6(1)
N(2)	27(2)	22(1)	28(2)	4(1)	4(1)	4(1)
N(3)	18(1)	34(2)	21(1)	-4(1)	2(1)	2(1)
N(4)	48(2)	72(3)	30(2)	5(2)	14(2)	-13(2)
C(1)	40(3)	28(3)	25(3)	-4(2)	12(3)	-3(3)
C(1')	29(6)	27(6)	33(7)	3(5)	11(6)	4(5)
C(1S)	63(4)	50(3)	53(3)	-4(3)	0(3)	-5(3)
C(2)	56(3)	31(2)	66(3)	-6(2)	37(3)	-5(2)
C(2S)	87(5)	74(4)	47(3)	-2(3)	21(3)	22(4)
C(3)	79(4)	40(3)	34(2)	15(2)	-12(2)	-25(3)
C(4)	84(4)	32(2)	38(2)	0(2)	-24(3)	15(2)

	U^{11}	U^{22}	U^{33}	U^{23}	U^{13}	U^{12}
C(5)	19(3)	19(3)	22(3)	0(3)	3(2)	-3(3)
C(5')	28(6)	64(9)	24(5)	-4(6)	13(4)	-12(6)
C(6)	21(3)	29(4)	21(3)	-11(3)	9(3)	-8(3)
C(6')	22(5)	42(8)	29(7)	0(6)	0(5)	-3(7)
C(7)	39(2)	54(3)	25(2)	6(2)	9(2)	-5(2)
C(8)	49(3)	44(2)	24(2)	-7(2)	13(2)	-21(2)
C(9)	68(3)	79(4)	28(2)	-5(2)	20(2)	-26(3)
C(10)	62(3)	56(3)	29(2)	-1(2)	24(2)	-19(2)
C(11)	65(3)	42(3)	56(3)	13(2)	20(3)	-4(2)
C(12)	53(3)	62(4)	68(4)	17(3)	21(3)	-1(3)
C(13)	69(4)	66(4)	50(3)	-3(3)	29(3)	-24(3)
C(14)	88(5)	55(3)	70(4)	-24(3)	52(4)	-21(3)
C(15)	67(4)	71(4)	58(3)	-21(3)	41(3)	-9(3)
C(16)	26(2)	36(2)	24(2)	0(2)	5(1)	9(2)
C(17)	28(2)	32(2)	24(2)	1(2)	5(1)	3(2)
C(18)	64(3)	82(4)	28(2)	-14(2)	1(2)	25(3)
C(19)	81(4)	60(3)	33(2)	-5(2)	17(3)	-12(3)
C(20)	25(2)	30(2)	51(2)	-7(2)	7(2)	-7(2)
C(21)	32(2)	32(2)	32(2)	-2(2)	7(2)	-8(2)
C(22)	43(3)	32(2)	64(3)	-7(2)	13(2)	-12(2)
C(23)	43(3)	43(3)	108(5)	-12(3)	22(3)	-17(2)
C(24)	31(2)	31(2)	36(2)	5(2)	-1(2)	-2(2)
C(25)	21(2)	39(2)	35(2)	6(2)	7(2)	4(2)
C(26)	31(2)	20(2)	34(2)	1(1)	5(2)	4(1)

Table S6. Hydrogen coordinates (×10^4) and isotropic displacement parameters ($Å^2$×10^3).

	**x**	**y**	**z**	**U (eq)**
H(1A)	1364	-584	1156	37
H(1B)	2667	-780	1468	37
H(1'A)	2689	-810	1486	36
H(1'B)	3413	-836	2556	36
H(4)	2100(30)	1380(20)	50(30)	36
H(2A)	2642	-1474	2654	56
H(2B)	1536	-1729	1842	56
H(2BC)	2334	-1767	2511	56
H(2BD)	1368	-1434	1642	56
H(3A)	1256	-1343	4129	71
H(3B)	2174	-1816	3857	71
H(4A)	3364	-928	4279	75
H(4B)	2722	-822	4990	75
H(5A)	3189	1063	3705	25
H(5B)	4109	449	4290	25
H(5'A)	4041	460	4413	45
H(5'B)	3864	-410	4008	45
H(6A)	3925	443	2678	28
H(6B)	3710	-405	3025	28
H(6'A)	4072	282	2899	40
H(6'B)	3365	998	3068	40
H(7A)	2271	1240	1759	48
H(7B)	1401	678	1041	48
H(9A)	2744	1189	-1089	69
H(9B)	3438	450	-561	69
H(11)	5320	611	-302	65
H(12)	6943	1354	79	74

	X	y	z	U (eq)
H(13)	6897	2679	416	71
H(14)	5218	3266	317	78
H(15)	3593	2511	-46	72
H(16A)	1652	351	4950	36
H(16B)	2189	1089	4629	36
H(18A)	4642	1180	7165	74
H(18B)	3853	772	7650	74
H(19A)	3631	2357	7053	88
H(19B)	4190	2096	8102	88
H(19C)	2889	1948	7573	88
H(20A)	-277	-1352	1888	44
H(20B)	-294	-1343	2911	44
H(22A)	-411	-3787	2915	57
H(22B)	-715	-3730	1822	57
H(23A)	-2589	-3513	1630	99
H(23B)	-2305	-3508	2717	99
H(23C)	-2196	-4301	2207	99

Table S7. Torsion angles.

Torsion Angles (°)			
Re(1)-N(1)-C(1)-C(2)	-50.5(5)	C(3)-N(2)-C(20)-C(21)	-62.9(5)
Re(1)-N(1)-C(1')-C(2)	11.5(12)	C(4)-N(3)-C(5)-C(6)	64.2(7)
Re(1)-N(1)-C(6)-C(5)	-21.3(7)	C(4)-N(3)-C(5')-C(6')	125.8(11)
Re(1)-N(1)-C(6')-C(5')	43.7(13)	C(4)-N(3)-C(16)-C(17)	59.8(5)
Re(1)-N(1)-C(7)-C(8)	-172.5(3)	C(5)-N(3)-C(4)-C(3)	-121.4(6)
Re(1)-N(2)-C(2)-C(1)	-10.8(5)	C(5)-N(3)-C(16)-C(17)	-69.9(5)
Re(1)-N(2)-C(2)-C(1')	26.9(9)	C(5')-N(3)-C(4)-C(3)	-123.5(7)
Re(1)-N(2)-C(3)-C(4)	-39.3(6)	C(5')-N(3)-C(16)-C(17)	-44.3(8)
Re(1)-N(2)-C(20)-C(21)	-177.0(3)	C(6)-N(1)-C(1)-C(2)	70.1(7)
Re(1)-N(3)-C(4)-C(3)	-6.8(7)	C(6)-N(1)-C(7)-C(8)	59.7(5)
Re(1)-N(3)-C(5)-C(6)	-54.5(6)	C(6')-N(1)-C(1')-C(2)	119.9(11)
Re(1)-N(3)-C(5')-C(6')	14.2(14)	C(6')-N(1)-C(7)-C(8)	77.7(7)
Re(1)-N(3)-C(16)-C(17)	-178.7(3)	C(7)-N(1)-C(1)-C(2)	-167.0(5)
O(1S)-C(1S)-C(2S)-F(1S)	49.4(8)	C(7)-N(1)-C(1')-C(2)	-125.6(8)
O(1S)-C(1S)-C(2S)-F(2S)	170.4(6)	C(7)-N(1)-C(6)-C(5)	106.4(6)
O(1S)-C(1S)-C(2S)-F(3S)	-71.6(8)	C(7)-N(1)-C(6')-C(5')	160.4(11)
O(2S)-C(1S)-C(2S)-F(1S)	-133.8(7)	C(8)-N(4)-C(9)-C(10)	-76.8(7)
O(2S)-C(1S)-C(2S)-F(2S)	-12.8(9)	C(9)-N(4)-C(8)-O(1)	4.4(7)
O(2S)-C(1S)-C(2S)-F(3S)	105.3(8)	C(9)-N(4)-C(8)-C(7)	-176.4(4)
N(1)-C(1)-C(2)-N(2)	43.3(7)	C(9)-C(10)-C(11)-C(12)	177.5(5)
N(1)-C(1')-C(2)-N(2)	-26.5(13)	C(9)-C(10)-C(15)-C(14)	-177.0(5)
N(1)-C(7)-C(8)-O(1)	-16.9(6)	C(10)-C(11)-C(12)-C(13)	-0.2(9)
N(1)-C(7)-C(8)-N(4)	164.0(4)	C(11)-C(10)-C(15)-C(14)	-0.6(8)
N(2)-C(3)-C(4)-N(3)	32.9(9)	C(11)-C(12)-C(13)-C(14)	-1.2(9)
N(2)-C(20)-C(21)-O(2)	-12.7(7)	C(12)-C(13)-C(14)-C(15)	1.7(9)
N(2)-C(20)-C(21)-O(3)	168.8(4)	C(13)-C(14)-C(15)-C(10)	-0.8(9)
N(3)-C(5)-C(6)-N(1)	52.7(8)	C(15)-C(10)-C(11)-C(12)	1.1(8)

Torsion Angles (°)			
N(3)-C(5')-C(6')-N(1)	-40.0(17)	C(16)-N(3)-C(4)-C(3)	115.4(6)
N(3)-C(16)-C(17)-O(4)	-5.3(6)	C(16)-N(3)-C(5)-C(6)	-169.7(5)
N(3)-C(16)-C(17)-O(5)	175.0(4)	C(16)-N(3)-C(5')-C(6')	-118.5(10)
N(4)-C(9)-C(10)-C(11)	127.2(5)	C(17)-O(5)-C(18)-C(19)	156.6(5)
N(4)-C(9)-C(10)-C(15)	-56.4(7)	C(18)-O(5)-C(17)-O(4)	5.1(7)
C(1)-N(1)-C(6)-C(5)	-136.1(5)	C(18)-O(5)-C(17)-C(16)	-175.1(4)
C(1)-N(1)-C(7)-C(8)	-63.2(5)	C(20)-N(2)-C(2)-C(1)	111.4(5)
C(1')-N(1)-C(6')-C(5')	-72.3(14)	C(20)-N(2)-C(2)-C(1')	149.1(8)
C(1')-N(1)-C(7)-C(8)	-35.2(8)	C(20)-N(2)-C(3)-C(4)	-157.4(5)
C(2)-N(2)-C(3)-C(4)	77.9(7)	C(21)-O(3)-C(22)-C(23)	179.0(4)
C(2)-N(2)-C(20)-C(21)	62.1(5)	C(22)-O(3)-C(21)-O(2)	-3.3(6)
C(3)-N(2)-C(2)-C(1)	-125.6(5)	C(22)-O(3)-C(21)-C(20)	175.2(4)
C(3)-N(2)-C(2)-C(1')	-87.9(9)		

6. References

(1) Braband, H.; Imstepf, S.; Benz, M.; Spingler, B.; Alberto, R. Combining bifunctional chelator with (3 + 2)-cycloaddition approaches: Synthesis of dual-function technetium complexes. *Inorg. Chem.* **2012**, *51* (7), 4051-4057. DOI: 10.1021/ic202212e.
(2) Hoerres, R.; Hennkens, H. M. 1,4,7-Triazacyclononane-based chelators for the complexation of [^{186}Re]Re- and [^{99m}Tc]Tc-tricarbonyl cores. *Inorg. Chem.* **2023**, *62* (50), 20688-20698. DOI: 10.1021/acs.inorgchem.3c01934.
(3) Alberto, R.; Egli, A.; Abram, U.; Hegetschweiler, K.; Gramlich, V.; Schubiger, P. A. Synthesis and reactivity of $[NEt_4]_2[ReBr_3(CO)_3]$. Formation and structural characterization of the clusters $[NEt_4][Re_3(\mu_3\text{-}OH)(\mu\text{-}OH)_3(CO)_9]$ and $[NEt_4][Re_2(\mu\text{-}OH)_3(CO)_6]$ by alkaline treatment. *J. Chem. Soc., Dalton Trans.* **1994**, (19), 2815-2820. DOI: 10.1039/DT9940002815.

Present Addresses:

[#]R.H.: Oak Ridge National Laboratory, 1 Bethel Valley Road, Oak Ridge, TN, 37830, United States

[$]R.K.: Curia Global, 21 Corporate Circle, Albany, NY, 12203, United States
